# Photocatalytic effect of gold-zinc oxide composite nanostructures for the selective and controlled killing of antibiotic-resistant bacteria and the removal of resistant bacterial biofilms from the body

**DOI:** 10.1186/s40580-025-00488-z

**Published:** 2025-05-14

**Authors:** Jongjun Park, Tae Hui Bae, Su Yong Kim, Seongeun Park, Yonghyun Choi, Masayoshi Tanaka, Jiwon Kim, Jaehee Jang, Jihyuk Yang, Hee-Young Lee, Tagbo H. R. Niepa, Shin Hyuk Kang, Jonghoon Choi

**Affiliations:** 1 School of Integrative Engineering, Chung-Ang University, Seoul, Republic of Korea; 2 Feynman Institute of Technology, Nanomedicine Corporation, Seoul, 06974 Republic of Korea; 3 Department of Plastic and Reconstructive Surgery, Chung-Ang University Gwangmyeong Hospital, Gwangmyeong-si, Gyeonggi-do 14353 Republic of Korea; 4 Department of Plastic and Reconstructive Surgery, Chung-Ang University Hospital, Chung-Ang University College of Medicine, Seoul, 06974 Republic of Korea; 5 Biomedical Research Institute, Chung-Ang University Hospital, Seoul, 06974 Republic of Korea; 6 Department of Chemical Science and Engineering, Institute of Science Tokyo, 4259 Nagatsuta-cho, Midori-ku, Yokohama-shi, Kanagawa 226-8503 Japan; 7 Department of Chemical Engineering, Kumoh National Institute of Technology, Gumi, 39177 Republic of Korea; 8 Department of Chemical Engineering, Carnegie Mellon University, Pittsburgh, PA USA; 9 Department of Biomedical Engineering, Carnegie Mellon University, Pittsburgh, PA USA

**Keywords:** Antibacterial, Antibiofilm, Photocatalyst, Nanoparticles, Antibiotic resistance

## Abstract

Infections involving antibiotic-resistant bacteria have become a major problem. Pathogenic bacteria use mechanisms such as drug target bypass, target modification, and biofilm formation to evade treatment. To respond to these problems, antibacterial research using metal and metal oxide nanoparticles is currently active. Nanoparticles treat bacterial infections through reactive oxygen species generation or antibacterial ion release. However, their application has faced problems related to human compatibility, as they react non-specifically, targeting both mammalian and bacterial cells. In addition, ZnO nanoparticles show low antibacterial activity against Gram-negative bacteria. Thus, the demand for antibacterial substances with enhanced specificity and improved efficacy is increasing. We bound gold to the surface of ZnO nanoparticles, enabling photocatalytic and photothermal actions through visible light irradiation. To improve bacterial specificity, Concanavalin A (Con A), a lectin that can specifically target bacterial membrane lipopolysaccharides, was conjugated with the nanoparticles. We showed that Con A-conjugated Au/ZnO nanoparticles (Au/ZnO-Con A) exhibit photocatalytic and photothermal effects under white light, enhancing their antibacterial ability, and through enhanced specificity, increased antibacterial and anti-biofilm abilities were confirmed. The developed particles showed the potential to alleviate antibiotic resistance in a bacterial skin infection model, presenting a new platform for treating bacterial infections.

**Graphical Abstract:**

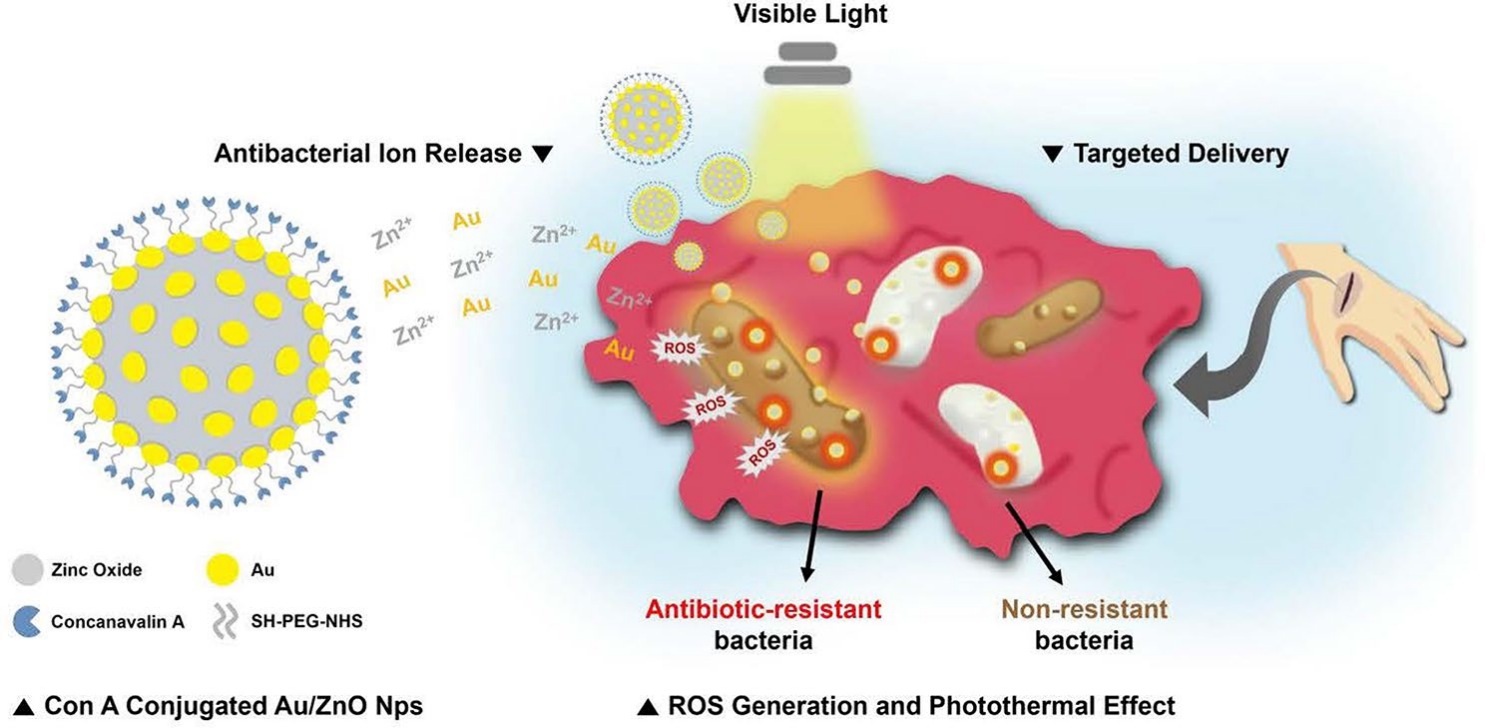

**Supplementary Information:**

The online version contains supplementary material available at 10.1186/s40580-025-00488-z.

## Introduction

Bacterial infections, fueled by antibiotic resistance, pose a major risk to humanity [1]. The indiscriminate use and misuse of antibiotics have led to the development of resistant strains, such as methicillin-resistant *Staphylococcus aureus*, and more recently, multi-drug resistant (MDR) strains have begun to emerge [2, 3]. The most common place where antibiotic-resistant bacteria are encountered is in hospitals, where antibiotics are most commonly used, and infections with multi-resistant bacteria can have dangerous consequences for patients with underlying medical conditions [4]. Most bacteria form biofilms that enhance the vitality of bacterial cells and protect them from the environment [5]. Biofilms are a highly challenging healthcare burden, severely complicating the treatment of bacterial infections by reducing the penetration and efficacy of antibiotics [6]. Additionally, biofilm formation in wounds caused from surgery or infections interferes with natural wound healing process and can lead to chronic wounds [7]. In patients with underlying medical conditions, chronic wounds can be devastating and it can cause variety of complications, including diabetic ulcers in patients with diabetes [8].

Various classes of antibiotics are now commercially available and are widely used around the world. They induce bacterial death through a multiple mechanisms, including inhibition of cell walls, proteins, and nucleic acid synthesis [9]. Antibiotics that target bacteria using specific mechanisms lose their efficacy because bacteria possess various defense strategies, such as efflux pumps, target bypass, target modification, and horizontal or vertical gene transfer [10]. Pathogens that survive drug treatment can transfer drug-resistant genes to other bacterial species, accelerating the spread of antibiotic resistance, and making it difficult to control [11].

Infections involving antibiotic-resistant bacteria are a challenge for existing antibiotic therapies and require the development of alternative approaches to mitigate them [12]. Among these, nanomaterial-based therapies using metal- or carbon-based nanoparticles and antimicrobial peptides have received much attention for their promising outcomes [13–15]. Due to their unique size and physical properties, they offer effective countermeasures against bacterial infections that can evade conventional antibiotic treatments. For instance, in a previous study assessing bimetallic nanoparticles, Jang et al. [16] demonstrated the antibacterial and antibiofilm abilities of silver and copper nanoparticles that release antimicrobial ions. The developed nanocomposites showed antibacterial activity comparable to conventional antibiotics and the antibiofilm effect was confirmed using microfluidic channels. Additionally, in vivo experiments in a rat wound model with *Pseudomonas aeruginosa* (*P. aeruginosa*) infection showed the potential clinical applications of the nanocomposite.

Zinc oxide (ZnO) nanoparticles are FDA-approved materials known for their biocompatibility and high antimicrobial activity [17]. Bacterial cells are particularly sensitive to Zinc ions (Zn^2+^), which induce bacterial cell death by various means, including the release of antimicrobial ions, generation of reactive oxygen species (ROS), and physical disruption of cell membranes [18–20]. However, despite ZnO nanoparticles showing a promising antibacterial effect against Gram-positive bacteria, they are less effective against Gram-negative bacteria [21, 22]. The lipopolysaccharide-containing outer membrane of Gram-negative bacteria hinders the permeation of Zn^2+^ ions, increasing the survival rate of bacterial cells [23, 24]. To address this, one could leverage the photocatalytic properties of nanomaterials. ZnO nanoparticles have the ability to absorb light in the ultraviolet (UV) region and are already used in many commercial sunscreens [25]. Therefore, under UV light irradiation, they transfer electrons, triggering photocatalytic activity. This process generates free radicals, which can be utilized to treat bacterial infection [26–28]. However, utilizing ZnO’s photocatalytic properties in actual clinical environments is challenging because it requires UV light irradiation, which is harmful to the human body [29]. Another drawback is that, like other metal particles, ZnO nanoparticles bind non-specifically to human and bacterial cells in wound sites. This may lead to direct particle toxicity, placing restrictions on the concentration of the nanoparticles used for treatment [30, 31].

In research focusing on harnessing the photocatalytic and antimicrobial properties of ZnO, there has been a growing body of work on using visible light as an alternative to UV light in phototherapy [32–34]. Zinc oxide particles are semiconductors with a large band-gap energy, and electrons in the valence band must be transferred to the conduction band using a high-energy source to achieve photocatalytic effects [35–37]. The electrons are transferred to the conduction band and the positive holes (h^+^) present in the valence band encounter oxygen and water, respectively, generating free radicals. To make this possible through visible light irradiation, the high band gap energy of ZnO must be reduced, and transition metals can be used to accomplish this [38, 39]. When synthesized together with an oxidizing metal, such as gold, the lowered band gap energy of the bimetallic combination allows for photocatalytic effects even with visible light [40]. In this study, gold–zinc oxide bimetallic (Au/ZnO) nanoparticles were synthesized utilizing gold and ZnO nanoparticles. Gold was selected among various metal particles due to its biocompatibility, stability, ease of modification, and photothermal properties. Gold nanoparticles are approved by the FDA and considered safe to use in biomedical applications. They exhibit strong colloidal stability and can be easily functionalized via interactions with thiol groups. Importantly, their photothermal effect under light irradiation induces localized heating, which may contribute to bacterial stress and membrane damage. Using gold, we expected photothermal and photocatalytic effects in the visible light region with low toxicity [41–43].

Concanavalin A (Con A) is a lectin, a class of proteins that exhibit sugar-specific binding. Con A is a monomeric 273-residue glycoprotein (MW = 27 kDa) which has a secondary structure composed of β-sandwich strands that requires metal ions for proper folding, stability, and function [44]. It is one of the most stable lectins in terms of physicochemical properties, withstanding pH levels of 5–8 and temperatures up to 60 °C. Also, α-D-Man, α-D-Glu, and β-D-Fru are known to bind specifically to Con A [45]. Lectins have been widely used in studies of leukemia, liver cancer, breast cancer, and among others, as glycan-binding probes that can recognize specific glycan structures [46, 47]. More recently, Con A has also been used in the field of bacterial detection, taking advantage of the fact that it specifically binds to sugars present on the surface lipopolysaccharides (LPSs) of Gram-negative bacteria [48, 49].

**Fig. 1:**
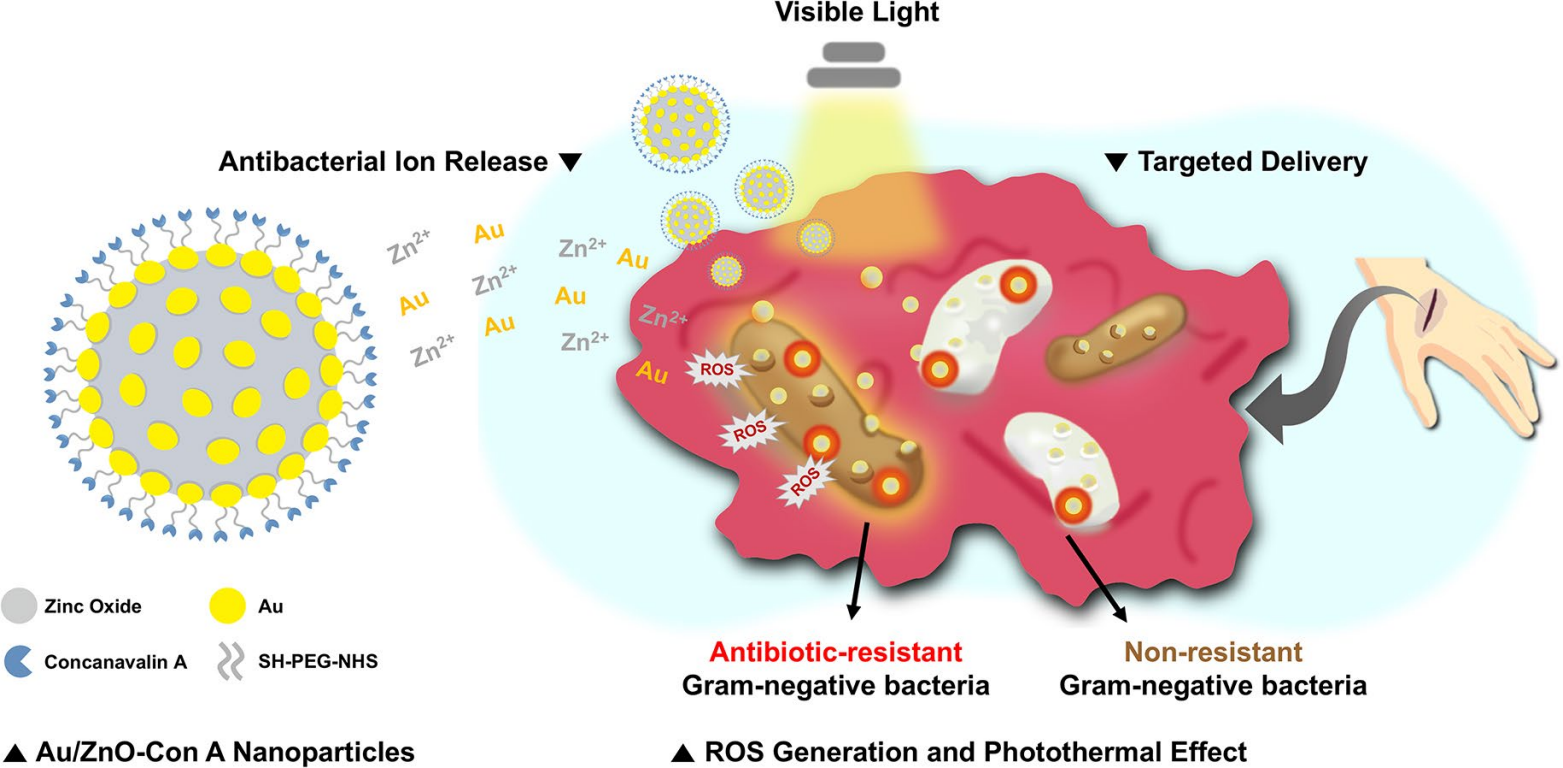
Illustration of an Au/ZnO-Con A nanoparticle (left) and its antibacterial mechanism of action through bacteria targeting and visible light irradiation (right)

To address the non-specific binding, cytotoxicity, and insufficient antibacterial activity against Gram-negative bacteria of ZnO nanoparticles, this study synthesized Au/ZnO nanoparticles conjugated with Con A (Au/ZnO-Con A; Fig. 1). The synthesized nanoparticles were irradiated with white LED light to assess increases in antibacterial and antibiofilm activity, resulting from ROS generation and photothermal effects. Con A, which can specifically bind to bacteria, was conjugated to the surface of the particles to eliminate non-specific reactions, and solve the problem of cytotoxicity.

## Material and method

### Materials

Several materials were used without further purification. Of these, zinc acetate, sodium hydroxide, gold (III) chloride trihydrate, sodium borohydride, concanavalin A, and crystal violet solution were purchased from Sigma-Aldrich (St. Louis, MO, USA). Ethyl alcohol, absolute, was purchased from Daejeong Reagent (Seoul, Korea). Thiol-polyethylene glycol-N-hydroxy-succinimide (SH-PEG-NHS) was purchased from Nanocs Inc. (NY, USA). Calcein AM, ethidium homodimer (EthD-1), and LIVE/DEAD *Bac*Light Bacterial Viability Kits were purchased from Invitrogen (Thermo Fisher Scientific Inc, MA, USA). Tryptic soy agar (TSA), tryptic soy broth (TSB), and antibiotic-antimycotic (A/A, 100×) were purchased from BD difco (NJ, USA). Calcein Red-AM was purchased from BioLegend (CA, USA). Human skin fibroblast cell line CCD-986sk cells were purchased from the Korea Cell Line Bank (Seoul, Korea), and RPMI 1640 medium was purchased from Welgene (Gyeongsan, Korea). Cell Counting Kit-8 (CCK-8) assay kits were purchased from DoGenBio (Seoul, Korea). Bacterial strains including *P. aeruginosa* and *Acinetobacter baumannii* (*A. baumannii*) were provided by Chung-Ang University Hospital (Seoul, Korea).

### Synthesis of ZnO nanoparticles

Prior to the synthesis of Au/ZnO bimetallic nanoparticles, ZnO nanoparticles were synthesized using the precipitation method [50]. One hundred mg of cetyltrimethylammonium bromide (CTAB) and 280 mg of zinc acetate were added to 40 mL ethanol. The temperature was then raised to the boiling point and 0.5 M NaOH solution was slowly added. The solution was stirred for 1 h as the color changed from colorless to white due to precipitation. A washing process was carried out using distilled water (DW), and the Zn(OH)_2_ was obtained by centrifugation. Finally, the pallet was dried in a vacuum to obtain ZnO particles.

### Synthesis of Au/ZnO bimetallic nanoparticles

To produce Au/ZnO nanoparticles, a deposition-precipitation method was applied to adsorb gold nanoparticles onto the metal oxides [51]. First, a 0.1 M NaOH solution was added to 2 mL of a 6.35 mM HAuCl_4_ solution and stirred for 15 min. The HAuCl_4_ was hydrolyzed to form a light-yellow colored gold hydroxide (Au(OH)_3_) solution. Next, a 30 mg mL^− 1^ ZnO solution was added, and the mixture was stirred at 100 °C on a heated stirrer for 1 h. After the Au(OH)_3_ nanoparticles were adsorbed onto the surface of the ZnO, they were washed with DW, centrifuged at 10,000 g at 25 ℃ for 15 min, and suspended in 1 mL of DW. This solution of ZnO with adsorbed Au(OH)_3_ was labeled “Au-seeded ZnO.” Finally, 1 mL of the Au-seeded ZnO solution was mixed with 2 mL of the HAuCl_4_ solution and 100 µL of 10 mM sodium citrate was added. The solution was reduced with a 6.6 mM NaBH_4_ solution, stirred for 20 min, washed with DW, and dried in vacuum, yielding the Au/ZnO nanoparticles.

### Conjugation of Au/ZnO to Con A

First, Au/ZnO particles were PEGylated through the specific adsorption reaction of gold and thiol groups, and the surface functional groups were surface modified with NHS groups that can bind amines using SH-PEG-NHS linker [52, 53]. A 5 mg mL^− 1^ solution of Au/ZnO and a 10 mg mL^− 1^ solution of SH-PEG-NHS were mixed in a 1:1 ratio and stirred for 24 h. The solution was washed with DW and spun down by centrifugation to eliminate the unconjugated PEG linkers. Then, the particles were dispersed in 1 mL of MES (2-(N-morpholino)ethanesulfonic acid) buffer. The Au/ZnO-SH-PEG-NHS solution and 2 mg mL^− 1^ Con A were mixed in a 1:1 ratio and stirred for 24 h. The Au/ZnO-Con A nanoparticles were washed in DW and spun down by centrifugation and finally dispersed in 1 mL DW. For experiments requiring fluorescence analysis, conjugation was performed using Con A-FITC.

### Nanoparticle characterization

To confirm the synthesis and morphology of Au/ZnO nanoparticles, they were analyzed using transmission electron microscopy (TEM; JEOL JEM-F200) at an accelerating voltage of 200 kV. The Au/ZnO nanoparticles were suspended at a concentration of 0.2 mg mL^− 1^, and samples were prepared by dispensing 10 µL onto a carbon film 300 mesh nickel grid. An energy-dispersive X-ray spectroscopy (EDS) analysis was performed using the same equipment, and an elemental identification of the synthesized particles was performed. The crystal structures of ZnO and Au/ZnO nanoparticles were determined using X-ray diffractometry (XRD, New D8-Advance). The size and zeta potential of the nanoparticles were measured by dynamic light scattering (DLS, Zetasizer Pro), and the particle concentration was determined by nanoparticle tracking analysis (NTA, NanoSight LM10). Absorbance spectra were measured using a microplate reader (BioTeK, Synergy H1) by dispensing a 0.5 mg mL^− 1^ solution of nanoparticles into a 96-well plate. To quantify the Con A lectin conjugated on the Au/ZnO nanoparticles, the BCA protein assay kit (ThermoFisher, Pierce) was used according to the manufacturer’s protocol. To confirm the binding of the SH-PEG-NHS linker to the Au/ZnO nanoparticles, the ZnO, Au/ZnO, and Au/ZnO-Con A peaks were measured using Fourier transform infrared (FT-IR, Alpha II, Bruker).

### Cytotoxicity tests

The cytotoxicity of Au/ZnO-based nanoparticles was confirmed using a CCK-8 assay. A normal human dermal fibroblast CCD-986sk cell line was maintained in RPMI 1640 medium (10% FBS, 1% A/A) at 37 ℃ and 5% CO_2_. The wells of a 96-well plate were seeded with 1 × 10^4^ cells per well and incubated for 24 h. Samples were diluted at various concentrations in cell media and applied to the cells. After 24 h, the cells were washed with Dulbecco’s Phosphate Buffered Saline (DPBS) and diluted 10-fold with the counting kit medium. After the reaction for 2 h, the supernatant was collected, and the absorbance was measured at 450 nm using a plate reader. Empty medium was used as a negative control and 1% Triton X-100 was used as a positive control. For light irradiation experiments, 35 mm dishes were used instead of 96-well plates.

Cytotoxicity was further assessed by staining living and dead cells using Calcein AM and EthD-1 dye. Fibroblast cells were cultured as described above, seeded at 3 × 10^5^ cells in 35 mm confocal dishes, and incubated for 24 h. Samples were treated with various concentrations of Au/ZnO-Con A particles and incubated for another 24 h after. The samples were then removed and washed with DPBS. In 10 mL of medium, 20 µL of 2 mM EthD-1 and 5 µL of 4 mM Calcein AM were mixed together and 1 mL was added to each dish. After 20 min of reaction, fluorescence images were taken using a STELLARIS 5 confocal microscope (LEICA, Munich, Germany).

### Photocatalytic activity tests

A methylene blue degradation test was performed to confirm the generation of free radicals by Au/ZnO-Con A nanoparticles due to the photocatalytic effect. A 20 µg mL^− 1^ methylene blue solution was prepared, and the sample solutions of nanoparticles at different concentrations were added and stirred for 30 min under dark conditions to achieve adsorption–desorption equilibrium and then irradiated with white light for 20 min. The particles were removed using a centrifuge and the absorbance spectra (400–800 nm) and absorbance at 660 nm were measured using a microplate reader. A mixture of Au/ZnO-Con A particles and 0.3% H_2_O_2_ solution was used as positive control. The degradation efficiency of the photocatalyst was determined by the equation

$$\:\left(\text{\%}\right)\:\text{d}\text{e}\text{g}\text{r}\text{a}\text{d}\text{a}\text{t}\text{i}\text{o}\text{n}=\:\frac{{A}_{0}-{A}_{\text{t}}}{{A}_{0}}\:\times\:\:100$$

where *A*_0_ and *A*_t_ are the absorbance at 660 nm of the negative control and treatment solutions, respectively.

### Photothermal performance tests

One mL aliquots of a 5 × 10^8^ particles mL^− 1^ Au/ZnO-Con A solution were dispensed into Eppendorf tubes (E-tube). These were irradiated for up to 30 min with a white light LED floodlight (30 W) and thermal images were recorded and peak temperatures were measured via a infrared camera. A ZnO solution at the same concentration and DW were used as controls. To evaluate thermal stability, tests alternating between light irradiation and darkness were performed. One mL aliquots of a 5 × 10^8^ particles mL^− 1^ Au/ZnO-Con A solution were irradiated with white light for 30 min and then held in darkness for 10 min while the temperature was measured every 10 min using a thermal imaging camera. This process was repeated three times. To explore how temperature varied with particle concentration, Au/ZnO-Con A solutions of different concentrations were prepared, with pure DW as the control. These were irradiated for a total of 30 min while temperature measurements were taken at 10-minute intervals.

### Ion release measurement

The ion release of Au/ZnO-Con A nanoparticles was observed using inductively coupled plasma atomic emission spectroscopy (ICP-AES, OPTIMA 8300). Nanoparticle solutions were prepared at concentrations of 1, 3, and 5 × 10^8^ particles mL^− 1^ and maintained under shaking conditions. After 3, 6, 12, and 24 h, only the supernatant was removed after centrifugation at 10,000 g and 25 °C for 15 min and used to analyze the released metal ions.

### Bacteria binding affinity tests

The bacteria-specific binding ability of Au/ZnO-Con A particles was evaluated using one strain each of *P. aeruginosa* and *A. baumannii*. Both were isolated patient samples from Chung-Ang University Hospital. They were cultured at 37 °C using TSA and TSB media. Bacterial solutions at a concentration of 1 × 10^9^ CFU mL^− 1^ were centrifuged at 3000 g for 15 min and washed with DPBS. Fixation was performed using a 4% paraformaldehyde (PFA) solution for 30 min at room temperature. After another washing with DPBS, the bacterial solutions were treated with Au/ZnO-Con A with FITC-conjugated Con A at a concentration of 1 × 10^9^ particles mL^− 1^. Con A-FITC and Au/ZnO-BSA were used as controls, with FITC conjugated to BSA. After 2 h of treatment, samples were washed twice with DPBS and 5 µL was aliquoted onto a glass slide with mounting medium and viewed using a confocal microscope.

For the conjugation of FITC to BSA, a 1 mg mL^− 1^ BSA solution and a 1 mg mL^− 1^ FITC solution were prepared using borate buffer. For the FITC solution, 2% dimethyl sulfoxide (DMSO) was used. Then, 400 µL of the BSA solution and 40 µL of the FITC solution were mixed and reacted for 2 h at 37℃. The reaction was then washed by centrifugation at 14,000 g for 15 min using a centrifugation filter. This process continued until the filtered solution was clear and a standard curve was obtained using the fluorescence values.

### Antibacterial tests

100 µL of each type of bacteria (1 × 10^6^ CFU/mL) was inoculated into TSB medium and incubated along with negative control. For the treatments, 100 µL of different concentrations of ZnO, Au/ZnO-Con A, and 1% A/A, used as a positive control, were applied. Additionally, Au/ZnO-Con A treatments were performed with and without a 20-minute light treatment at 3 cm above the sample. After 24-hour incubation at 37℃, 100 µL of the samples were diluted and spread on TSA solid medium. The colonies on the plate were counted after incubation at 37℃ for 24 h. All experiments were run in triplicate.

For the bacterial live and dead cell assays, the *Bac*light bacterial viability kit was utilized following the manufacturer’s protocol. Bacterial solutions at 1 × 10^9^ CFU mL^− 1^ were treated with particle solutions at 4 × 10^8^ particles mL^− 1^ and incubated for 24 h. First, appropriate amounts of 3.34 mM SYTO 9 dye and 20 mM propidium iodide (PI) solutions were mixed in a 1 to 1 ratio. For every 1 mL of bacteria, 3 µL of the dye mixture was added and the reaction was carried out in the dark for 15 min. Then 5 µL of the stained bacterial suspension was mounted on a glass slide and observed using a confocal microscope.

### Biofilm inhibition test

To determine the antibiofilm effect of Au/ZnO-Con A particles in the presence and absence of light, the crystal violet staining method was used to confirm biofilm inhibition. Each bacterium (100 µL) was inoculated into TSB medium and incubated along with negative control. 100 µL of different concentrations of Au/ZnO-Con A particles were applied, and half the Au/ZnO-Con A treatments also received a light treatment, and 1% A/A solution was used as a positive control. After 24 h of incubation at 37 °C, the samples were washed with DPBS to remove suspended bacteria. The plates were then fixed with 99.9% methanol for 20 min. After removing the supernatant, the sections were completely fixed for 30 min at room temperature and stained for 5 min with 0.1% crystal violet solution. For biofilm quantification, the plates were again washed with DPBS, and then a 70% ethanol solution was added, and absorbance was measured at 590 nm.

### In vivo wound healing tests in a mouse model

Experiments were performed according to protocols approved by the institutional animal care and usage committee (IACUC) of Chung-ang University (CHUNG-ANG IACUC 202301020138, January 2024). We aimed to assess the therapeutic efficacy of bacteria-targeting nanoparticles with light irradiation in a mouse model of skin infection induced by MDR *P. aeruginosa*. The bacterial strain used in the experiment showed resistance to penicillin, cephalosporin, and trimethoprim/sulfamethoxazole, exhibiting non-susceptibility in more than three antimicrobial categories, which designates it as an MDR bacteria [54].

Twenty-four 11-week-old male black mice (C57BL/6NTac, 22–25 g, DBL, Korea) were obtained and allowed to acclimate for one week. The laboratory environment was maintained at an ambient temperature of 22 ± 2°C with a 12-hour light-dark cycle. Water and feed were provided ad libitum. Successful establishment of the model was ensured by removing full-thickness skin and exposing the area to *P. aeruginosa* for 24 h [55]. Mice (*n* = 6 per group) were anesthetized with a 3:2 zoletil–rompun mix, and their backs were shaved within a 4 cm^2^ area. All wounds were cleansed with pure saline, and using a punch biopsy tool, a 5 mm full-thickness skin defect was created. A 0.5 mm silicone sheet splint with an 8 mm hole was placed over the wound. We anchored the splint with interrupted 6 − 0 nylon sutures to ensure positioning. Using silicone sheet splints facilitates the maintenance of a transparent occlusive dressing and helps reduce the difference in the process of contraction between murine and human wounds, thus aiding in replicating human wound healing more accurately [56].

To induce bacterial infection, 50 µL of a bacterial solution containing 1 × 10^6^ CFU mL^− 1^ was administered to the wound, followed by dressing with OpSite Flexifix (Smith & Nephew, Watford, England) to create biofilm conditions for 24 h. The wound was then drained of excess bacterial solution and re-covered with OpSite Flexifix [57]. The next day, the presence of a *P. aeruginosa* biofilm was confirmed using the handheld MolecuLight i: X (MolecuLight Inc., Toronto, Canada) fluorescence imaging device, which is capable of visualizing red or cyan fluorescence emitted by bacteria [58]. *Pseudomonas* spp. uniquely produces cyan-fluorescing siderophores known as pyoverdines, which manifest as cyan-fluorescing patterns on the fluorescence imaging device. Mice were divided into four groups (*n* = 6), and each group was administered either PBS (100 µL), tobramycin ointment, Au/ZnO-Con A (100 µL containing 4 × 10^8^ particles mL^− 1^), or Au/ZnO-Con A with a light treatment. The light treatment consisted of exposure to white light for 20 min immediately after the Au/ZnO-Con A treatment.

Treatment was administered on days 1 and 5, and wounds were photographed on days 0, 1, 3, 5, and 8 to assess changes in wound size and healing progression. Wound size was determined by averaging three measurements per wound using calipers. All the mice were sacrificed on Day 8 and bacteria from the wounds were collected using cotton swabs for bacteria spreading on the final day to quantitatively compare the remaining bacteria in each group after treatment.

Histological evaluations were performed on day 8 to assess healing qualities. For the histological analysis, plantar skin samples were removed from the mice’s backs. The samples were fixed with 4% formaldehyde and embedded in paraffin. Consecutive Sect. (10 μm thickness) were stained with hematoxylin-eosin (H&E).

### Statistical analysis

All data and graphs were processed using GraphPad Prism (v8.0.2, GraphPad Software Inc., La Jolla, USA). One-way ANOVAs followed by Dunnett’s multiple comparisons tests were performed to assess the statistical significance of differences between treatments and control. In figures, significant differences are indicated by asterisks: *, *p* < 0.05; **, *p* < 0.01; ***, *p* < 0.001; and ****, *p* < 0.0001. Non-significant values are represented as “ns.”

## Results and discussion

### Synthesis of Au/ZnO nanoparticles

**Fig. 2:**
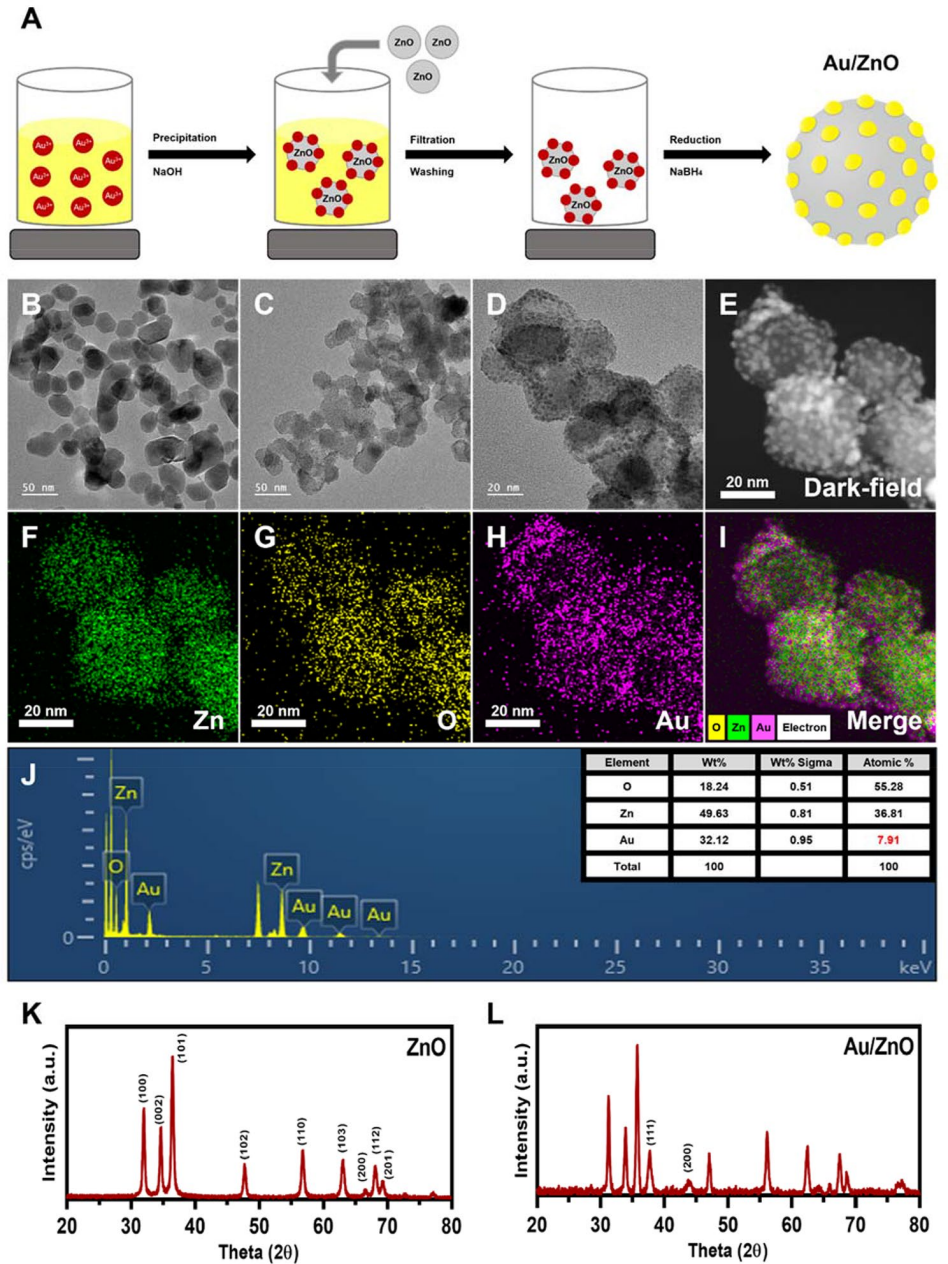
Characterization of Au/ZnO nanoparticles. (**A**) A schematic illustration of the Au/ZnO nanoparticle synthesis process. (**B**) A TEM image of ZnO nanoparticles. (**C**, **D**) TEM images of Au/ZnO nanoparticles at different magnifications. (**E**) A dark-field image of Au/ZnO nanoparticles. (**F**–**I**) EDS mapping images of Au/ZnO nanoparticles. (**J**) An EDX analysis of Au/ZnO nanoparticles. (**K**, **L**) XRD patterns of ZnO and Au/ZnO nanoparticles

Zinc oxide nanoparticles were synthesized via a double displacement reaction using a precipitation method and solutions of zinc acetate and NaOH. Zinc acetate was hydrolyzed to form Zn^2+^ ions, which were precipitated as Zn(OH)_2_ upon contact with NaOH. From this, ZnO particles were synthesized by condensation process under vacuum and high temperature (Figure S1). The synthesized ZnO nanoparticles were confirmed by TEM images (Fig. 2B).

Next, Au/ZnO nanoparticles were synthesized using the precipitation–deposition method, a method that allows the adsorption of transition metals onto the surface of metallic oxide particles (Fig. 2A). First, the HAuCl_4_ precursor was hydrolyzed with NaOH to produce Au(OH)_3_ seeds, which changed the color of the solution from yellow to yellowish white. In an aqueous environment, ZnO nanoparticles are positively charged, while Au seeds are negatively charged. Consequently, when ZnO nanoparticles were added, the Au seeds were adsorbed onto the surface of ZnO nanoparticles through the electrostatic interaction. The Au-seeded ZnO nanoparticles were then redispersed in HAuCl_4_ solution for growth and reduction, and then NaBH_4_ was used to obtain gold nanoparticles. According to literature, adsorbed gold content depends on serval factors such as concentration of the gold seed or the growth degree of the particles [59]. TEM images at different magnifications showed that the gold nanoparticles were successfully adsorbed onto the ZnO nanoparticles. By using uniform solid seeds, gold nanoparticles were synthesized equally well on the ZnO surface (Fig. 2C–E). The elements Zn, O, and Au were all identified by EDS mapping, and the elemental percentage of gold was confirmed to be about 7.91% by EDX analysis (Fig. 2F–J).

To analyze the crystal structures of ZnO and Au/ZnO particles, XRD crystalline peaks were produced (Fig. 2K, L). The crystalline peaks of ZnO confirmed the hexagonal ZnO phase (wurtzite structure) of the as-synthesized ZnO nanoparticles through the ZnO characteristic peaks (JCPDS No. 36-1415). In particular, the main 2θ values corresponding to the (1, 0, 0), (0, 0, 2), (1, 0, 1), (1, 0, 2), (1, 1, 0), (1, 0, 3), (2, 0, 0), (1, 1, 2), and (2, 0, 1) lattice planes were identified and showed obvious ZnO peaks. When the XRD peaks of Au/ZnO were compared with the ZnO peaks, all the existing ZnO peaks were confirmed, and in addition, the 2θ values corresponding to lattice planes (1, 1, 1) and (2, 0, 0) were unique peaks of gold particles, indicating the successful synthesis of Au/ZnO bimetallic nanoparticles (JCPDS No. 4-0783).

### Synthesis of Au/ZnO-Con A nanoparticles

**Fig. 3:**
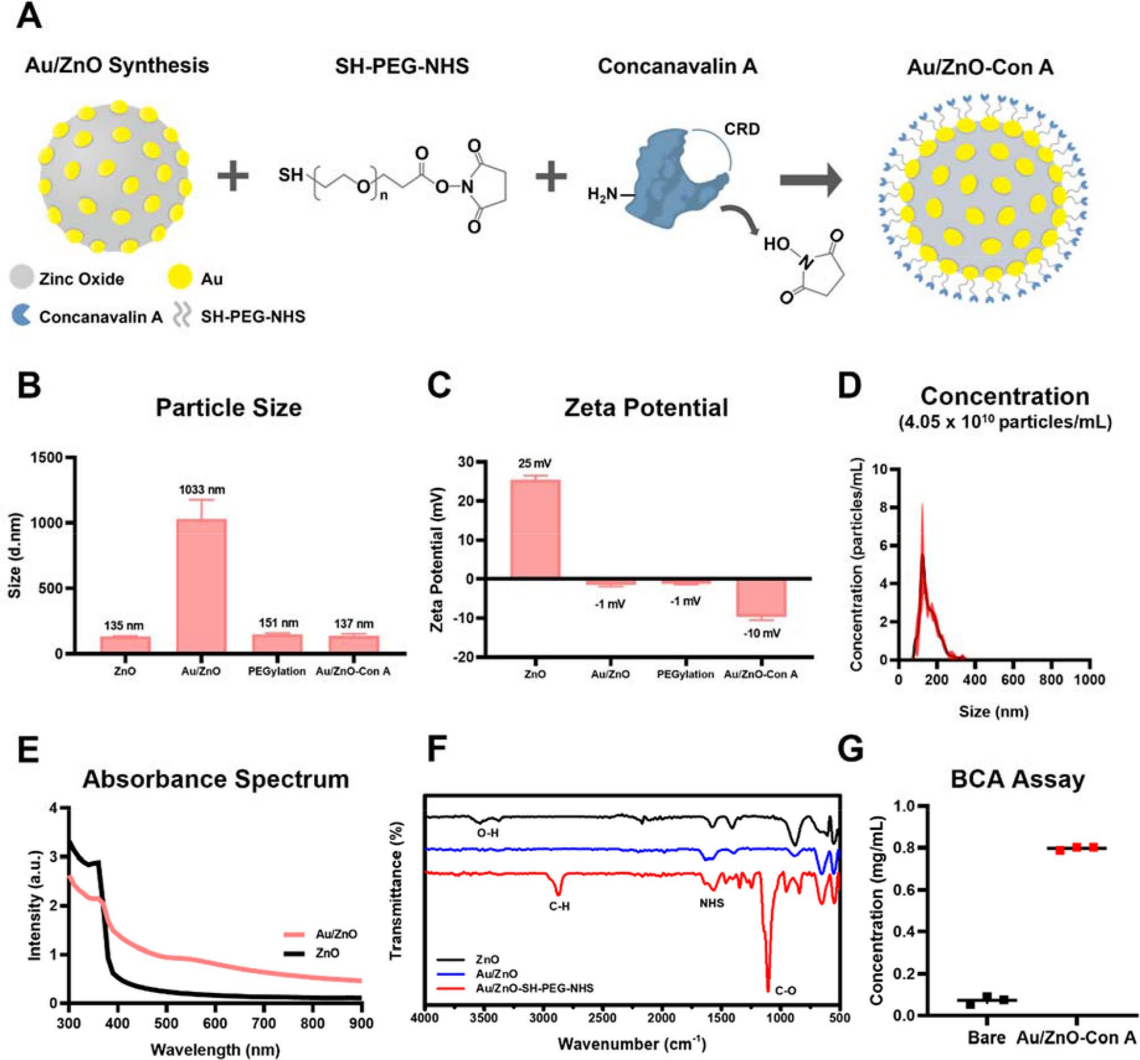
(**A**) A schematic illustration of the Au/ZnO-Con A preparation process. (**B**) The average particle size of different particles and (**C**) zeta potential, as determined by DLS. Error bars represent standard deviation for three independent analyses. (**D**) The Au/ZnO-Con A concentration, as determined by NTA. (**E**) The absorbance spectra of ZnO and Au/ZnO. (**F**) The transmittance FT-IR spectra of ZnO, Au/ZnO, and Au/ZnO-SH-PEG-NHS. (**G**) Conjugated Con A protein concentration calculated using BCA assay

To fabricate Au/ZnO-Con A nanoparticles, bare Au/ZnO bimetallic particles were first PEGylated via SH-PEG-NHS linker. The specific reaction of gold with the thiol group of the linker maintained the stability of the particles and exposed the NHS group at the outermost angle to react with the amine group of the lectin (Fig. 3A). The size distribution and zeta potential of the synthesized particles were measured using DLS (Fig. 3B, C). The average size of ZnO nanoparticles was found to be 135.2 ± 2.00 nm, confirming that the synthesized particles were nano sized. In the case of bare Au/ZnO nanoparticles, the zeta potential was almost neutral at − 1 mV due to the adsorption of gold nanoparticles, and the aggregation of the particles was confirmed by the increase in the size of the DLS phase. Conjugation strategy using SH-PEG-NHS linker was attempted first to maintain the stability of the particles and use direct reaction of NHS and amine group of Con A. Consequently, through the specific interaction between the Au present in the Au/ZnO nanoparticles and the thiol groups from SH-PEG-NHS linker, the distance between the particles was secured by PEG, which alleviated the aggregation phenomenon and allowed the size to return to the nano scale [60, 61]. Further conjugation of Con A lectin increased the stability of the particles, which was confirmed by a further decrease in size in the DLS phase. The final Au/ZnO-Con A particles showed a negative zeta potential due to the influence of Con A on the surface, which was expected to reduce non-specific binding to cells with negatively charged membranes. An NTA analysis confirmed the particle concentration and size of the synthesized Au/ZnO-Con A (Fig. 3D), finding that the particles were uniform in size with an average diameter of 146.2 ± 46.8 nm, similar to the size determined by DLS which was 136.6 ± 18.14 nm. The concentration of the synthesized Au/ZnO-Con A was found to be 4.05 × 10^10^ particles mL^− 1^, and all subsequent experimental concentrations with the particles were diluted according to NTA.

Using a plate reader, the absorption peak in the characteristic UV range (360 nm) of ZnO nanoparticles was identified in the absorption spectrum (Fig. 3E). In comparison, the Au/ZnO particles showed the absorbance peak of gold nanoparticles in addition to that of ZnO. The introduction of gold nanoparticles resulted in a broad absorbance in the visible region, which enabled the induction of photocatalytic and photothermal effects through visible light irradiation. Wide range of absorbance spectrum resulted from formation of small Au particles on the ZnO surface, along with variations in their size. This feature was adequate for future light irradiation where LED white light covers the full visible light spectrum. To confirm the PEGylation of Au/ZnO nanoparticles, an FT-IR analysis was performed (Fig. 3F). This technique investigates the characteristic stretching and bending of functional groups such as C-H bending or C-O stretching. According to FT-IR, the PEG peak and NHS peak of PEGylated Au/ZnO nanoparticles were identified. First, the peak at 557 cm^− 1^ corresponds to the metal-oxygen intrinsic stretching vibration, which was identified for ZnO, Au/ZnO, and Au/ZnO-Con A. For ZnO nanoparticles, O–H stretching peaks were identified at 3539 cm^− 1^ and 3376 cm^− 1^, which were not identified after the adsorption of gold nanoparticles. The peaks seen at 2877 cm^− 1^ and 1105 cm^− 1^ for Au/ZnO-SH-PEG-NHS particles represent C–H stretching and C–O stretching, respectively, confirming the presence of PEG on the particle surface. The 1567 cm^− 1^ and 1650 cm^− 1^ peaks represent N–O and C = O stretching, respectively, thus confirming the presence of the NHS functional groups. To verify the final nanoparticle product, Au/ZnO-Con A, the conjugation concentration was confirmed by the BCA assay, which can quantify proteins (Fig. 3G). First, Con A lectin and a BCA solution were used to create a standard curve, and then bare Au/ZnO and Au/ZnO-Con A particles were tested to quantify the amount of lectin conjugated to the particles. When 1 mg mL^− 1^ Con A was added and conjugated, 0.8 mg mL^− 1^ Con A was calculated using the standard curve. Therefore, it was confirmed that Con A protein was successfully conjugated to the nanoparticle through the amine-specific binding of Au/ZnO-SH-PEG-NHS particles, and the final yield was about 80% based on the input protein.

### Cytotoxicity test

**Fig. 4:**
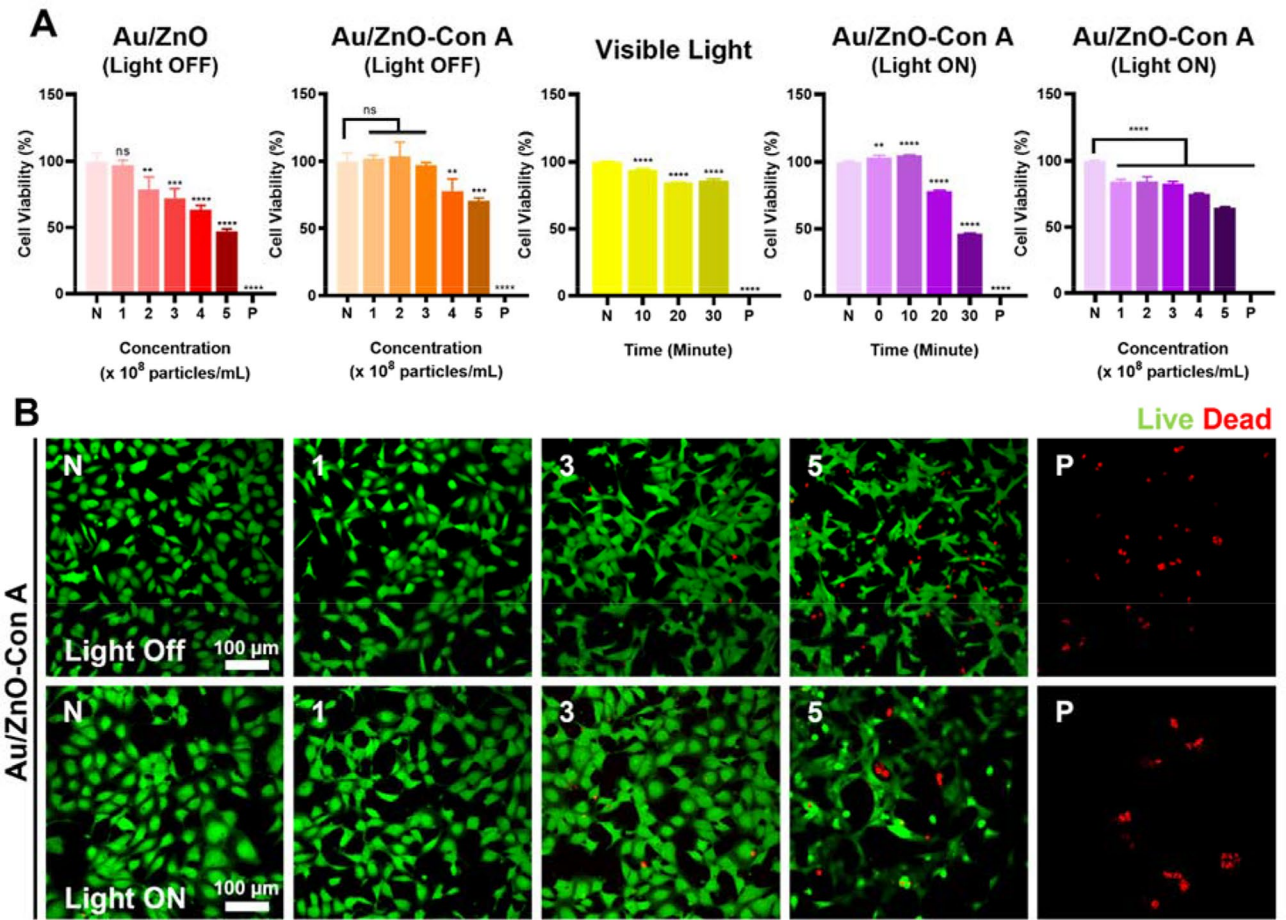
(**A**) Cytotoxicity evaluations using cell counting kit-8 (CCK-8). Au/ZnO nanoparticles and Au/ZnO-Con A nanoparticles were tested without light irradiation. Cell toxicity with different irradiation time was evaluated using a white light LED floodlight (30 W). Au/ZnO-Con A nanoparticles were tested with different irradiation time and concentrations with light irradiation. (**B**) Confocal images of live (green) and dead (red) cells after treatment with Au/ZnO-Con A particles at different concentrations in the presence or absence of light using CCD-986sk cell line cells. X-axis values in the upper left of the images represent treatments groups: “N” indicates “negative control” which was conducted without any particle treatment, numbers represent different concentrations used in particles × 10^8^ mL^− 1^, and “P” indicates “positive control” which 1% Triton X-100 was used. The first and second row experiments were conducted under identical conditions, with the only difference being the presence or absence of light

To evaluate the cytotoxicity of the synthesized particles, a CCK-8 assay was performed. A common skin fibroblast cell line, CCD-986sk, was used to simulate the skin environment surrounding the bacterial skin infection (Fig. 4A). The cytotoxicity of bare Au/ZnO nanoparticles became evident when tested at concentration of 4 × 10^8^ particles mL^− 1^, which corresponded to a reduction of cell viability to 63%. When Au/ZnO-Con A particles were used to treat the CCD-986sk cells, increased cell viability compared to the bare Au/ZnO was observed for all concentrations. There were with no significant difference between the untreated control and cells treated with Au/ZnO-Con A up to a concentration of 3 × 10^8^ particles mL^− 1^ (p value > 0.99). Even at the highest tested concentration 5 × 10^8^ particles mL^− 1^, cell viability remained at 71%, suggesting a low likelihood of nanoparticle-induced toxicity in the in vitro tests (ISO 10993-5). This suggests that the conjugation of the Con A lectin, leading to a negative zeta potential, reduced non-specific binding to cells. In addition, PEGylation and lectin conjugation resulted in more stable nanoparticles, which shielded the cells from the direct exposure and potential cytotoxicity of the Au/ZnO complex. The conjugated nanoparticles remained stable and did not aggregate unlike Au/ZnO nanoparticles, which size increased to the micro-scale (Fig. 3B). Although visible light irradiation alone showed relatively low toxicity up to the 30-minute treatment time, cell viability decreased by 46% as Au/ZnO-Con A (4 × 10^8^ particles mL^− 1^) were used along with the light irradiation for 30 min. Therefore, 20 min of irradiation was selected as the maximum irradiation time, and it was used for treatments in the remainder of the study. To determine the optimal concentration of Au/ZnO-Con A particles for further experiments, cytotoxicity was assessed after 20 min of irradiation. As a result, 4 × 10^8^ particles mL^− 1^, which maintained 75% cell viability, was chosen as the ideal concentration for biological studies (Fig. 4A).

The cytotoxicity evaluation of Au/ZnO-Con A nanoparticles using live and dead assay showed similar results as when tested using CCK-8 assay. The images taken by confocal microscopy presented qualitative view of Au/ZnO-Con A toxicity with and without light irradiation. Red cells which indicate dead cells began to appear at concentration 3 × 10^8^ particles mL^− 1^ for both light conditions and the number of dead cells increased as the concentration increased (Fig. 4B). At a concentration of 5 × 10^8^ particles mL^− 1^, toxicity became evident in both conditions, with noticeable cell shape damage and reduced cell density. A significant increase in dead cells was observed, which correlated with the trends seen in the CCK-8 assay. This allowed for the visual confirmation and validation of the previously obtained toxicity data.

### Au/ZnO-Con A antibacterial mechanisms

In the antibacterial effect of metal nanoparticles, bacterial cell damage due to ROS generation, photothermal effects, and metal ion release are known to be important mechanisms. Therefore, the developed Au/ZnO-Con A nanoparticles were designed to generate ROS, cause thermal stress, and release ions due to photocatalytic effects under visible light irradiation.

#### ROS production

**Fig. 5:**
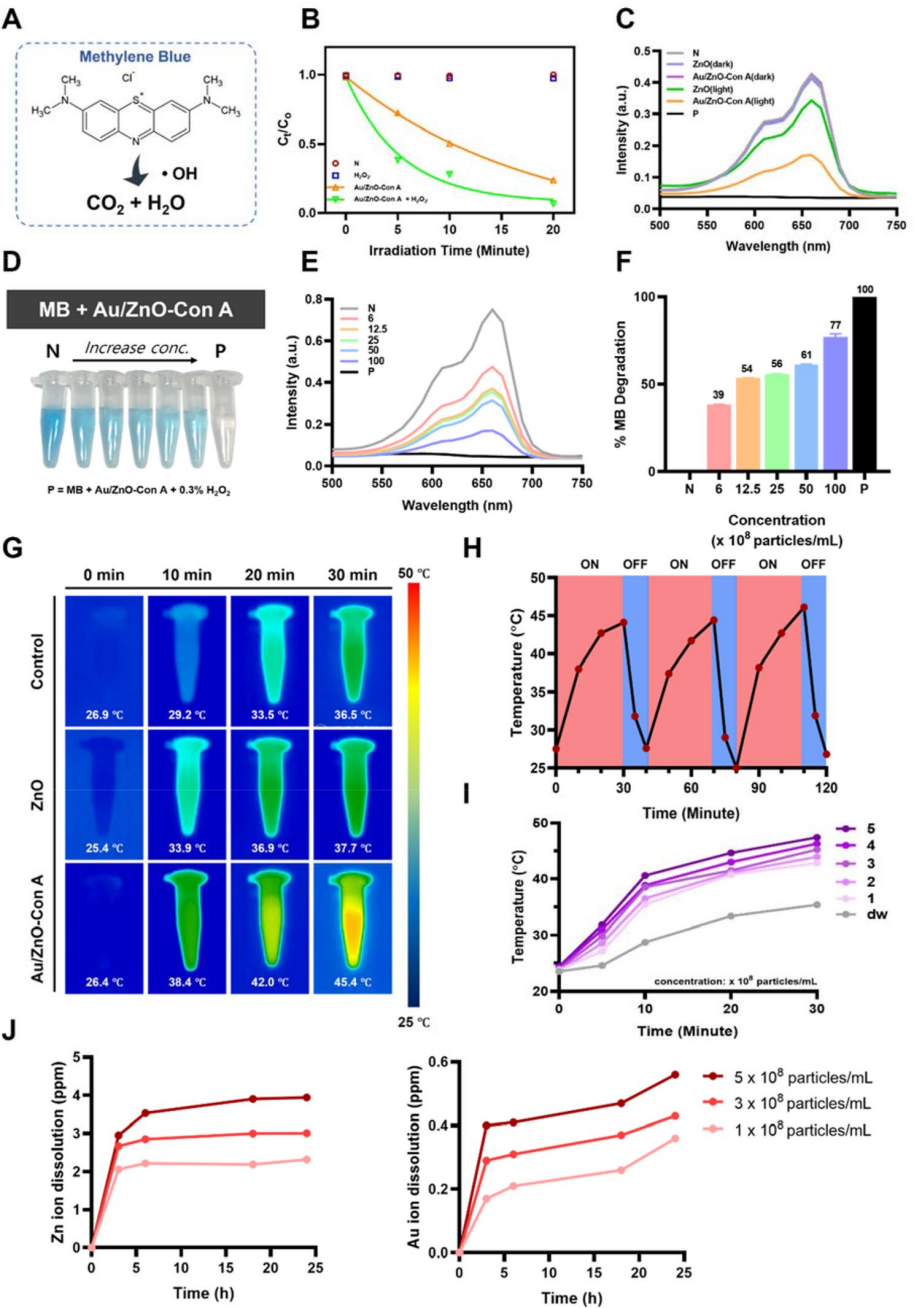
(**A**) Schematic illustrating methylene blue degradation via the hydroxyl radical. (**B**) The photocatalytic degradation rate of Au/ZnO-Con A and H_2_O_2_ under light irradiation (“N” represents “negative control” where only DW was used for control). (**C**) The UV–vis adsorption spectra of methylene blue solutions following degradation with different particles with and without light irradiation (“P” represents the “positive control”). (**D**) Image showing methylene blue color loss through degradation under different Au/ZnO-Con A nanoparticle concentration after 30 min of light irradiation. (**E**) UV–vis adsorption spectra of methylene blue solutions following degradation with different concentrations of Au/ZnO-Con A nanoparticles (in × 10^8^ particles mL^− 1^). (**F**) Methylene blue degradation rate at different Au/ZnO-Con A concentrations. (**G**) Infrared thermal images of the control, ZnO, and Au/ZnO-Con A solutions at different irradiation times. (**H**) Temperature change curve of a 5 × 10^8^ particles mL^− 1^ Au/ZnO-Con A solution over three white light (30 W) irradiation/darkness cycles. (**I**) Temperature change over time for different concentrations of Au/ZnO-Con A solutions during irradiation. (**J**) Time-dependent zinc and gold ion concentrations in aqueous solution, representing ions released from Au/ZnO-Con A nanoparticles at various concentrations

ZnO nanoparticles are known to exhibit photocatalytic effects, and photocatalysis in semiconductors is based on the ability to produce photogenerated electrons and holes in the valence and conduction bands, respectively, under light irradiation. As electrons in the valence band migrate to the conduction band, they reduce O_2_ present in aqueous solution to superoxide radicals, which subsequently form hydroxyl radicals through a series of reactions. In addition, the h + holes formed by the migrated electrons react with water and oxidize it to form hydroxyl radicals. However, ZnO is a material with a large band gap energy and must be irradiated with energy above UV light to generate free radicals. If gold, a transition metal that is considered harmless to the human body, is adsorbed on the surface of ZnO nanoparticles, the band gap energy can be reduced, and free radicals can be successfully produced even under visible light irradiation [62, 63]. In this study, the methylene blue degradation by hydroxyl radical was utilized to monitor the free radical generation of the Au/ZnO-Con A nanoparticles developed in this study using a plate reader under pH 7 conditions (Fig. 5A). First, Au/ZnO-Con A nanoparticles (1 × 10^10^ particles mL^− 1^) and 0.3% H_2_O_2_ were used to confirm the photocatalysis effect, and the results are shown in Fig. 5B. A significantly higher nanoparticle concentration was used in the ROS generation evaluation because methylene blue degradation serves as an indirect, macroscopic assessment of ROS production, requiring a larger scale concentration for detection. To verify Au/ZnO-Con A nanocomposite’s ROS generating ability, the test was conducted at higher concentrations than those used for cell-based assay [64]. This experiment focused on evaluating the ROS-generating capacity of the nanoparticles. In the group containing only DW and 0.3% H_2_O_2_, which was used as a negative control, no degradation of methylene blue was observed under 20 min of visible light irradiation. In the reaction with Au/ZnO-Con A, the degradation efficiency was significantly higher (76.3% at 20 min of irradiation, p value < 0.0001), and complete degradation was confirmed when H_2_O_2_ was added. H_2_O_2_ was used as a synergistic agent that enhances ROS mediated dye degradation. While H_2_O_2_ alone cannot degrade methylene blue, it can effectively react with the electrons from Au/ZnO-Con A nanocomposites. Electrons from the conduction band can interact with H_2_O_2_ to produce more hydroxyl radicals [65]. Complete degradation of methylene blue in the presence of H_2_O_2_ indicated that the electrons were successfully excited and actively contributed to ROS generation. Therefore, in subsequent particle-by-particle or concentration-by-concentration experiments, Au/ZnO-Con A + 0.3% H_2_O_2_ was used as the positive control. As shown in (Fig. 5C), under dark conditions, no degradation of methylene blue could be seen in the negative control, ZnO, and Au/ZnO-Con A groups. However, when irradiation with visible light for 20 min was added under the same condition, ZnO nanoparticles showed limited degradation, while Au/ZnO-Con A showed increased degradation. To confirm the exact degradation efficacy of the developed particles, the concentration-dependent degradation rate under light irradiation was determined (Fig. 5D–F). As the concentration of particles increased, the color of methylene blue became lighter, and when the organic dye degradation rate was calculated compared to the negative control, the highest tested concentration 1 × 10^10^ particles mL^− 1^ showed a 77% degradation rate. The methylene blue test indicated that the developed Au/ZnO-Con A nanoparticles could act as a photocatalyst and predicted a higher free radical release than ZnO nanoparticles under visible light irradiation.

#### Photothermal effects

Metal nanoparticles generate surface plasmon resonance (SPR) at the surface of the particles when irradiated with light, causing electrons and metal atoms to collide and release heat energy. Therefore, gold adsorbed on the surface of ZnO nanoparticles can not only lower the band gap energy but also produce photothermal effects under visible light irradiation [40]. To verify the photothermal effect in the synthesized Au/ZnO-Con A nanoparticles, a 1 mL sample solution was irradiated with a 30 W white light, and the temperature was measured using a thermal imaging camera (Fig. 5G). Distilled water and ZnO particles were used as negative controls in this experiment. All particle solutions were diluted to 5 × 10^8^ particles mL^− 1^ and subjected to light irradiation for up to 30 min. When the Au/ZnO-Con A nanoparticles were subjected to 30 min of light irradiation, a maximum temperature of 45.4 °C was observed. This temperature was about 9 ℃ greater than that of the negative control, DW, and 8 ℃ greater than that of ZnO particles. To evaluate the photothermal stability of Au/ZnO-Con A particles, the temperature was monitored through three light and darkness cycles (Fig. 5H). After 30 min of visible light irradiation, the temperature of the synthesized particles increased to about 45 °C, and after resting for 10 min, the temperature decreased back to room temperature. This same trend was observed over all 3 consecutive cycles, thus confirming the temperature increase in the presence and absence of light and demonstrating the photothermal stability of the particles. Finally, various concentrations of Au/ZnO-Con A were irradiated with visible light while their temperature was monitored (Fig. 5I). All the concentrations used showed an increased exothermic effect compared to the DW control, and the highest nanoparticle concentration, of 5 × 10^8^ particles mL^− 1^, was found to exotherm up to 47 ℃. Since the increased temperature reached up to 47 ℃, it is anticipated that this temperature alone is insufficient to eradicate bacteria. However, this temperature increase could enhance the synergistic antibacterial effects of metal ion release and reactive oxygen species production because the optimal growth temperature for most pathogenic bacterial strains is around 37 ℃ [66]. The heating itself might not be sufficient to completely eradicate bacteria, but localized temperature elevation at the bacterial membrane could induce enough cellular damage to enhance overall antibacterial efficacy. There has been reports where low-temperature photothermal therapy can have a negative influence on bacterial survival when particles are close by to the pathogenic cells [67, 68]. Therefore, the introduction of gold into the nanoparticle structure enabled a photothermic effect in Au/ZnO-Con A particles under visible light irradiation and confirmed that they can cause photothermic stress to bacterial cells.

#### Ion release

Various metals, ZnO, Cu, Ag, Au, etc., have been studied for their antimicrobial properties, and among the various identified antimicrobial mechanisms, antimicrobial metal ion release plays a very important role [69]. Inside microorganisms, metal ions can impair metal transport mechanisms and cause internal stress to the cell [70]. Furthermore, zinc ions in particular play an important role in human physiology and are actively utilized in wound healing, including cell membrane repair and cell proliferation. When we observed the release of metal ions in aqueous solution using three different nanoparticle concentrations, we recorded 3.93 and 0.56 ppm for zinc (Zn^2+^) and gold (Au^3+^), respectively, at the highest concentration (5 × 10^8^ particles mL^− 1^) (Fig. 5J). Such concentration of Zn^2+^ ions released falls into the antibacterial range reported in other literature [71]. Based on the magnitudes of ion release, we believe that zinc ions had more influence on the antibacterial ability than gold ions.

### Bacteria binding affinity test

Metal particles such as Au/ZnO can damage bacterial cells by generating ROS, heating, and releasing antimicrobial ions, as mentioned above, but excessive amounts can also adversely affect normal human cells. In this study, we introduced Con A, a lectin that can target Gram-negative bacteria, to endow the particles with bacteria-specific capabilities and minimal cytotoxicity to human cells. Con A is a mannose- and glucose-specific protein that can target LPSs on Gram-negative bacterial cells, providing a promising localized antimicrobial therapy.

The bacterial binding ability of Au/ZnO-Con A was evaluated using Gram-negative bacteria, *P. aeruginosa* and *A. baumannii* strains. All bacteria were DNA stained with Hoechst dye and treated with Con A, Au/ZnO-Con A, and Au/ZnO-BSA to determine binding by confocal microscopy via green intensity. Con A-FITC and BSA-FITC were used during conjugation to make the particles fluoresce green. Con A was treated as a positive control to confirm the specific binding of Con A lectin to LPSs present on the surface of Gram-negative bacteria. Au/ZnO-BSA nanoparticles were used as a negative control to confirm that the reaction between Au/ZnO-Con A and LPS was not by non-specific binding.

**Fig. 6:**
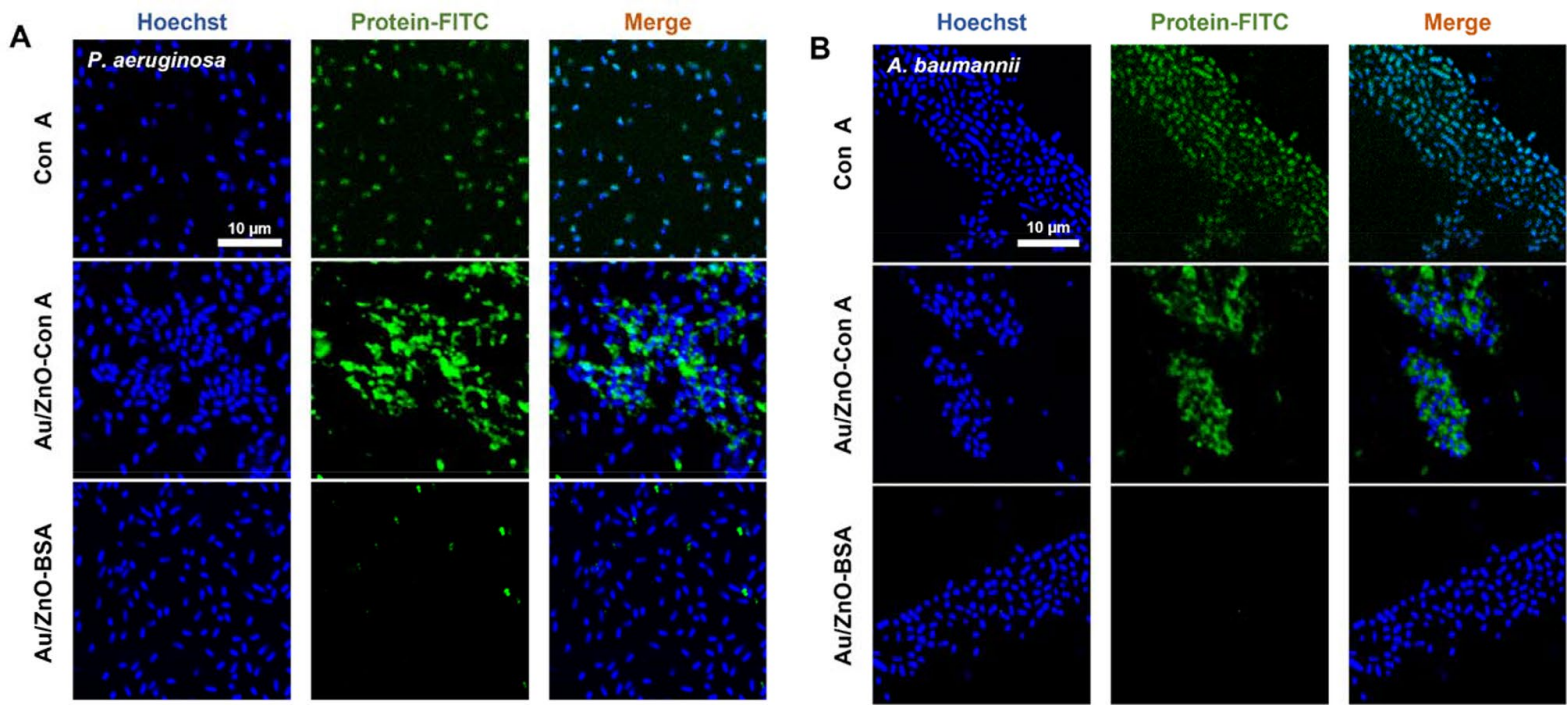
Bacteria binding affinity tests. Confocal microscope images of Hoechst and protein-FITC (Con A-FITC or BSA-FITC) staining of (**A**) *P. aeruginosa* and (**B**) *A. baumannii* after treatment with Con A, Au/ZnO-Con A, or Au/ZnO-BSA

For the *P. aeruginosa* strain, all bacteria were stained using Hoechst stain (Fig. 6A). In the group treated with Con A alone, specific binding between Con A lectin and bacteria was confirmed, and Con A bound to all the bacteria stained in blue. This showed that Con A can target bacteria through specific bindings to mannose and glucose in their LPSs. Treatment of Au/ZnO-Con A particles also confirmed the specific binding of particles to the bacteria. The binding of the Con A on the surface of the particles to the bacteria-induced aggregation, and the presence of particles between the bacteria confirmed their ability to capture the bacteria. In contrast, in the case of Au/ZnO-BSA, it was difficult to see the green signal of the particles, indicating that the Au/ZnO-Con A particles bind to bacteria through a Con A–mannose-specific reaction rather than a non-specific reaction. The *A. baumannii* strain showed a similar trend to that seen in *P. aeruginosa*. The addition of Con A to the fabricated nanoparticles allowed them to target both strains of Gram-negative bacteria. This result indicated the non-discriminatory ability of Au/ZnO-Con A to bind to LPS-producing strains, distinguishing it from antibodies that react only to a single species. By using lectins to target bacteria, the delivered nanoparticles can effectively generate heat and oxygen radicals, enabling localized antibacterial treatments.

### Antibacterial and antibiofilm effects

To evaluate the antibacterial ability of the fabricated bacteria-targeting particles, bacterial viability was checked by using the micro broth dilution method where minimum inhibitory concentration (MIC) of an antibacterial agent can be determined through serial dilution. All strains used were clinical samples provided by Chung-Ang University Hospital and all were tested to confirm they were MDR bacteria (Table S1). Different concentrations of ZnO and Au/ZnO-Con A particles were tested for their antibacterial ability. In the case of Au/ZnO-Con A, all concentration treatments were tested with and without a 20-minute white light irradiation treatment following the addition of the particle solution.

**Fig. 7:**
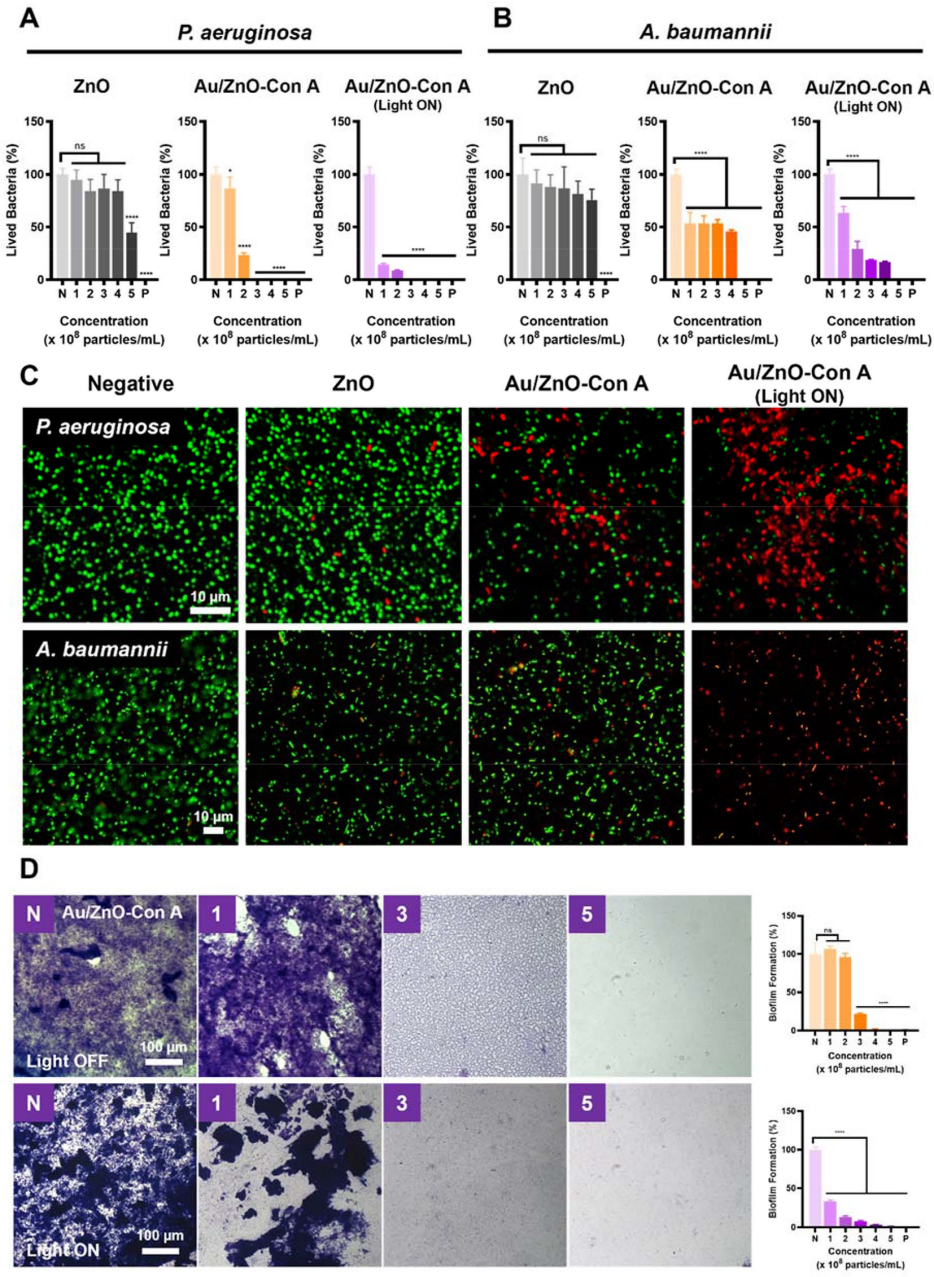
Antibacterial tests against (**A**) *P. aeruginosa* and (**B**) *A. baumannii* bacteria strains using ZnO and Au/ZnO-Con A nanoparticles and Au/ZnO-Con A nanoparticles with a light treatment (light ON). (**C**) Live and dead cell assays using different particle treatments: green stain marks all bacteria, and red stain indicates dead cells. (**D**) Antibiofilm effect of Au/ZnO-Con A on *P. aeruginosa* without (top) or with (bottom) light through crystal violet staining. Bar plots on the right show a quantitative analysis of the effects of Au/ZnO-Con A on biofilms as seen in the images on the left. 1% A/A solution was used as positive control for antibacterial and antibiofilm tests

When the *P. aeruginosa* strain was treated with ZnO nanoparticles, minimal antibacterial activity was seen at concentrations below 4 × 10^8^ particles mL^− 1^ (Fig. 7A). It is plausible that under these conditions, Zn^2+^ ions, known for their antibacterial activity, might be released at concentration tolerated by *P. aeruginosa*. In contrast, Au/ZnO-Con A nanoparticles showed increased antibacterial activity, which was attributed to the specific binding of Con A to bacteria. MIC of Au/ZnO-Con A nanoparticles against P. aeruginosa strain was found to be 3 × 10^8^ particles mL^− 1^ due to the induced aggregation of the bacteria and particles, as well as the localized presence of antimicrobial ions in the surrounding environment. Furthermore, the bactericidal effect of the nanoparticles increased when irradiated with white light due to the generation of heat and free radicals. When light was applied to Au/ZnO-Con A nanoparticles, the antibacterial effect significantly increased at low concentration (1 × 10^8^ particles mL^− 1^), with bacteria survival rate dropping from 87 to 14%. In the case of the *A. baumannii* strain, Au/ZnO-Con A nanoparticles showed higher antibacterial activity than ZnO single particles, and the killing effect was increased when combined with light irradiation (Fig. 7B).

The bacteria-specific targeting ability and enhanced antimicrobial activity upon light irradiation were also confirmed by a bacteria Live and Dead assay. For both the *P. aeruginosa* and *A. baumannii* strains, SYTO 9 dye was used to stain all bacteria green and PI dye was used to mark dead cells. When treated with the 4 × 10^8^ particles mL^− 1^ of each particle type, the highest killing effect was observed when Au/ZnO-Con A particles were combined with light irradiation (Fig. 7C). For both strains, the antibacterial effect was higher for Au/ZnO-Con A nanoparticles with Con A-mediated specific binding to bacteria, and the effect was enhanced by light irradiation due to the photothermal and photocatalytic effects. Au/ZnO-Con A nanocomposites showed greater bactericidal efficacy in *P. aeruginosa* compared to *A. baumannii*. This difference may be attributed to the more robust defense mechanisms of A. baumannii, which is known to exhibit enhanced tolerance to elevated levels of ROS and high-temperature stress [72]. Since the antibacterial effect of light and bacteria targeting was confirmed in both strains, subsequent anti-biofilm and in vivo animal model evaluations were performed using only the *P. aeruginosa* strain.

The anti-biofilm inhibitory ability of Au/ZnO-Con A nanoparticles was confirmed by crystal violet staining. When the sample was untreated, the *P. aeruginosa* strain formed biofilms in all 96 wells. When the particle treatments alone, without light irradiation, were used, no biofilm inhibition could be seen below a concentration of 2 × 10^8^ particles mL^− 1^ (Fig. 7D). At 3 × 10^8^ particles mL^− 1^, a change in biofilm morphology could be observed: the biofilm formed by the bacteria became thinner and began to show a grid-like structure. The inhibitory capacity was 22% compared to the control. At higher concentrations, no biofilm was visible.

Treatments combining the Au/ZnO-Con A particles and white light irradiation for 20 min confirmed a biofilm inhibition ability at low concentrations. At a concentration of 1 × 10^8^ particles mL^− 1^, 32% biofilm formation was observed, showing greatly increased antibiofilm ability compared to the Au/ZnO-Con A treatment without light. At higher concentrations, biofilms were mostly inhibited. This suggests that the biofilm-inhibiting ability of the nanoparticles was enhanced by the increased free radicals and heat generated by the light irradiation treatment. Our results revealed that light irradiation in conjunction with Au/ZnO-Con A nanoparticles was sufficient to inhibit biofilm formation without using high concentrations of particles. Low-concentration treatments can minimize cytotoxicity while allowing for the safe targeting of bacteria, killing them, and inhibiting their protective biofilm.

### Antibacterial effect of nanoparticles in vivo

Twenty-four mice were randomly divided into four groups, and there were no significant differences in wound size before starting treatment. Two mice from the negative control group, one from the tobramycin group, and one from the Au/ZnO-Con A group died, presumably due to acute *P. aeruginosa* infection or inability to withstand frequent anesthesia.

**Fig. 8:**
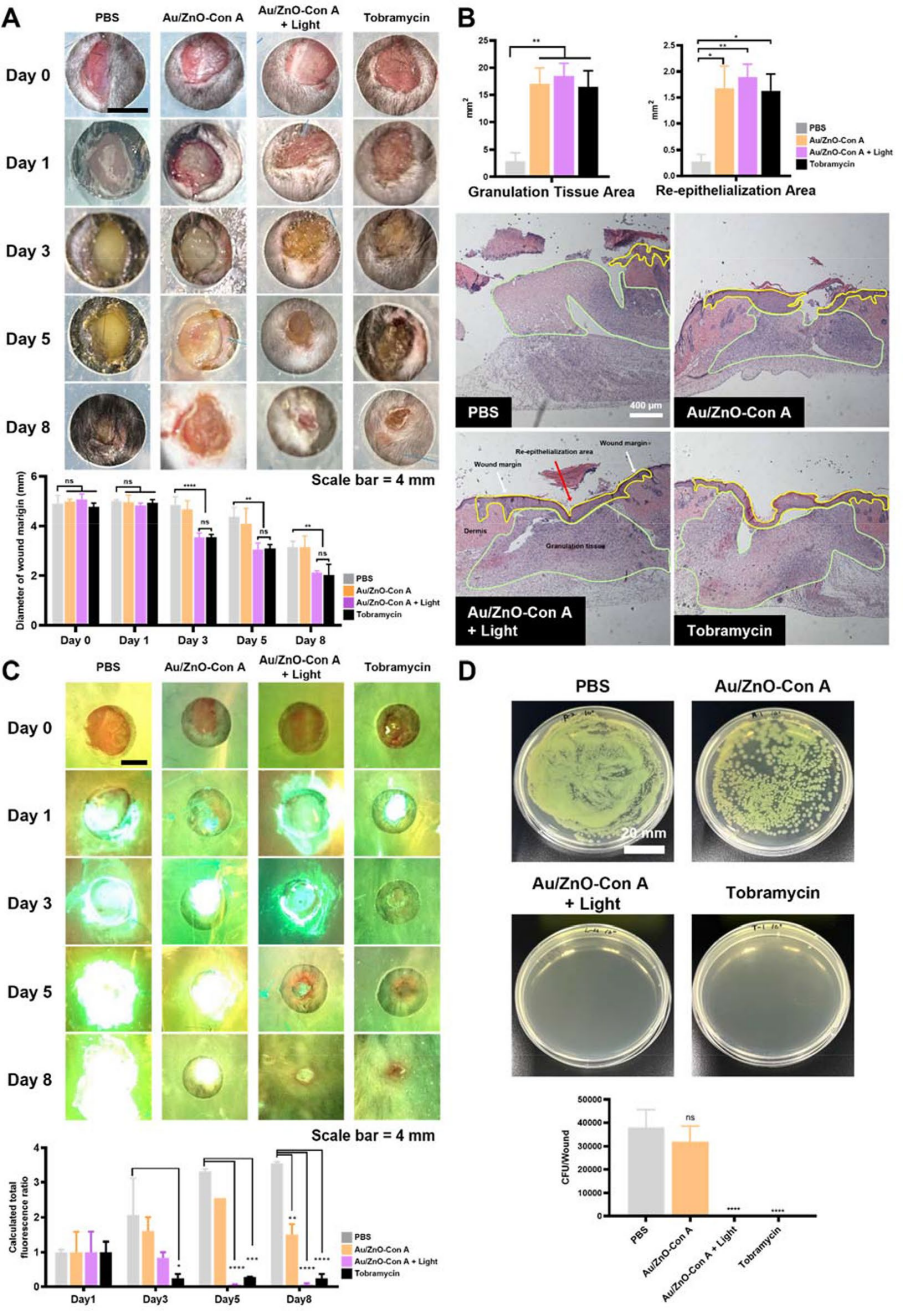
Treatment of *P. aeruginosa*-infected wounds on C57BL/6 mice using PBS (100 µL), tobramycin ointment, Au/ZnO-Con A (100 µL containing 4 × 10^8^ particles mL^− 1^), or Au/ZnO-Con A + Light (Au/ZnO-Con A in conjunction with a light treatment). (**A**) Representative photographs of infected wounds over time for PBS (negative control), Au/ZnO-Con A, Au/ZnO-Con A + Light, and Tobramycin groups (scale bar = 4 mm). Pictures were then taken using an iPhone 15. Wound areas over time for Data are presented as means ± SEM (*n* = 4), and statistical significance is denoted by asterisks: **, *P* < 0.01 and ****, *P* < 0.0001 (two-way ANOVA test). (**B**) A histological quantitative morphometric analysis including representative microscopic photos of each group at different time points using H&E staining (×4; scale bars = 400 μm). The wound margins (white arrows), granulation tissue areas (green lined area), and re-epithelialization areas (yellow lined area, B) were representatively marked. The granulation tissue area and re-epithelialization were quantitatively evaluated (above). Data are presented as means ± SEM (*n* = 4), and statistical significance is denoted by asterisks: *, *P* < 0.05 and **, *P* < 0.01 (One-way ANOVA test). (**C**) Wound imaging with a fluorescence imaging device (MolecuLight i: X). The calculated total fluorescence ratio for days 3, 5, and 8 represents the relative values calculated by dividing each result by the value on Day 1. Data are presented as means ± SEM (*n* = 2), and statistical significance is denoted by asterisks: *, *P* < 0.05; **, *P* < 0.01; ***, *P* < 0.001; and ****, *P* < 0.0001 (Two-way ANOVA test). (**D**) The numbers of *P. aeruginosa* CFUs after swabbing and spreading the wounds on day 8 following each treatment. Data are presented as means ± SEM (*n* = 4), and statistical significance is denoted by asterisks: ****, *P* < 0.0001 (One-way ANOVA test)

On day 3, macroscopic suppuration and inflammation were observed in the wounds of the negative control group and the Au/ZnO-Con A group (Fig. 8A). Analysis of wound size data showed that the Au/ZnO-Con A + Light group exhibited a reduced wound size compared to the negative control group on day 8, with the most significant difference observed on day 3 after the first treatment. This indicates that from the start of treatment, both the Au/ZnO-Con A + Light group and the Tobramycin group showed significantly superior ability to heal wounds.

Following a morphological assessment of all tissues, paraffin blocks were prepared, and histological analyses were performed only on the widest area of the wound. To evaluate wound recovery in each group, H&E staining was performed on the skin of the original wound area on day 8, executing a histological quantitative morphometric analysis using ImageJ.

For each wound, the granulation tissue area was traced at the superior aspect of the granulation tissue or the junction between the granulation tissue and epidermal area, with the inferior border traced between the adipose tissue or muscle and the inferior border of the granulation tissue. The re-epithelialization area was measured, with the last hair follicle of uninjured tissue on both sides as reference points (Fig. 8B; Au/ZnO-Con A + Light group, white arrows). When measuring areas, the scab was excluded.

The measurement results showed that the Au/ZnO-Con A, Au/ZnO-Con A with light, and the tobramycin groups all showed larger granulation tissue areas (Fig. 8B) than the PBS (negative control) group, However, only the Au/ZnO-Con A + Light group showed a significantly higher value compared to the PBS group. This indicates that Au/ZnO-Con A + Light dressing improved wound healing by promoting the formation of granulation tissue.

Regarding the re-epithelialization area, the Au/ZnO-Con A, Au/ZnO-Con A + Light, and the tobramycin groups all showed statistically significantly wider areas compared to the PBS group, with no significant differences observed among the other groups. Faster re-epithelialization often results in less scar tissue formation and a better cosmetic appearance of the healed wound.

On day 1, after 24 h of exposure to *P. aeruginosa*, biofilm formation was confirmed in all 24 wounds using MolecuLight i: X (Fig. 8C). For a quantitative evaluation of wound healing from a bacterial infection angle, bacterial spreading assays were conducted. In the Au/ZnO-Con A + Light group and Tobramycin group, no bacteria were detected (0 CFU mL^− 1^), while the remaining two groups showed approximately 3 × 10^4^ CFU mL^− 1^ (Fig. 8D). This indicates a significantly superior antibacterial effect in the Au/ZnO-Con A + Light group and the tobramycin group (Fig. 8C). MolecuLight i: X data showed that in the Au/ZnO-Con A group, the detection intensity of cyan fluorescence increased until day 5, but decreased on day 8, while in both the Au/ZnO-Con A + Light group and the tobramycin group, the intensity of fluorescence decreased over time after the initial infection, and by day 8, there was minimal cyan fluorescence remaining. In the PBS group, the intensity and area of fluorescence showed a progressively increasing trend (Fig. 8C).

In the in vivo experimental results, treatment with Au/ZnO-Con A did not show a level of wound healing comparable to that of the Au/ZnO-Con A + Light or the tobramycin group. In in vitro studies, when Au/ZnO-Con A was used without a light treatment, complete bacterial eradication was observed at concentrations of 3 × 10^8^ particles mL^− 1^ or higher, and when the visible light treatment was added, complete bacterial eradication was achieved at even lower particle concentrations. Unlike the immediate treatment conducted in the in vitro experiments, the in vivo tests were preceded by a 24-hour incubation period between bacteria injection and treatment initiation. Therefore, it is estimated that the bacterial concentration within the wound was higher at the start of treatment. Also, bacterial behaviors can have differences between in vitro and in vivo settings. In vivo environments often provide conditions that are more favorable for bacterial survival, showing differences in treatment responses [73]. After the 24-hour incubation period, the formation of bacterial colonies was confirmed using the non-invasive MolecuLight i: X to ensure the establishment of an appropriate wound infection model before proceeding with treatment. This method is promising, as it does not require swabbing and allows for infection confirmation. It is possible that the concentration of the Au/ZnO-Con A solution in the in vivo experiment was set too low, which may have prevented significant results in wound healing.

Furthermore, in terms of wound size and bacterial spreading, both the Au/ZnO-Con A + Light and the tobramycin groups showed similar effects. Histologically, the Au/ZnO-Con A + Light group exhibited significantly higher granulation tissue formation than the PBS group, but no significant difference was found between the Au/ZnO-Con A + Light and the tobramycin groups. However, it has been reported that topical antimicrobial formulations containing aminoglycosides can show adaptive cross-resistance and lead to infections progressing to multidrug-resistant *P. aeruginosa* [74]. When topical antimicrobial formulations are improperly used or overused, there is a possibility of progression beyond MDR to extensively drug-resistant (XDR) or pan-drug-resistant (PDR) bacteria, which can further worsen a patient’s prognosis [54, 74]. Considering this, the experimental result is promising as it demonstrates the potential of using Au/ZnO-Con A as a replacement for conventional antibiotics in the future. Additionally, the method of using Au/ZnO-Con A with visible light can be considered a novel approach, but optimization of a protocol for utilizing visible light therapy with Au/ZnO-Con A for clinical applications appears to be necessary.

## Conclusions

Antibiotic-resistant bacteria have various adverse effects on infections, including chronic wounds that do not heal. We have developed a technology that can effectively treat antibiotic-resistant bacterial infections through biocompatible nanoparticles we designed. To potentiate ZnO nanoparticles, which can compensate for the shortcomings of antibiotics, we synthesized Au/ZnO bimetallic nanoparticles and maximized the antibacterial effect through white light irradiation, taking advantage of the features of bimetallic nanoparticles. To improve the specificity of the bimetallic nanoparticles, which can also be toxic to normal animal cells, we developed Au/ZnO-Con A, a nanoparticle that can specifically bind to bacteria by conjugating Con A, a mannose and glucose-specific lectin that can target bacterial EPS, with the particles.

To confirm the maximized antimicrobial activity of the fabricated Au/ZnO-Con A nanoparticles, the photothermal and photocatalytic effects were examined. It was found that Au/ZnO-Con A particles were more exothermic than conventional ZnO nanoparticles when treated with visible light irradiation, confirming the possibility cell killing through heat stress. In addition, the photocatalytic role of the particles was confirmed through a methylene blue degradation assay, and it was found that they could degrade organic dyes through the generation of free radicals. When cytotoxicity was evaluated using CCD-986sk cell line cells, the toxicity of Au/ZnO-Con A nanoparticles was lower than that of bare Au/ZnO nanoparticles, and the maximum concentration and white light irradiation time that can be applied to normal cells was found. The bacteria-specific binding of Au/ZnO-Con A particles to Gram-negative bacteria, including *P. aeruginosa* and *A. baumannii* cells, was characterized and revealed that both strains could be targeted through Con A–bacteria binding using the particles. As a final in vitro test, the antibacterial and anti-biofilm effects were checked, it was confirmed that the targeting ability alone increased the antibacterial ability. In addition, the antibacterial and anti-biofilm ability was enhanced by the increase of free radicals generated by light irradiation and the photothermal effect. Lastly, a wound-healing mouse model in vivo experiment showed that the Au/ZnO-Con A treatment in conjunction with a light treatment produced results comparable to antibiotic treatments. In this study, the primary objective was to evaluate the overall antibacterial properties of the Au/ZnO-Con A nanocomposites. While variations in antibacterial efficacy were observed depending on the treatment concentration, both bacterial strains tested in vitro exhibited clear susceptibility. Future investigations will aim to further explore the antibacterial spectrum of the nanocomposite, including its effect against different bacterial species. The Au/ZnO-Con A nanocomposite demonstrated promising potential not only as an antibacterial agent, but also a material that may promote wound healing. While the primary focus of this study was on assessing the antimicrobial properties, nanocomposites under light irradiation exhibited faster healing compared to the control group. Investigations into the wound healing properties of Au/ZnO-Con A nanocomposite could provide important insights. In the future, this technology is expected to provide a new treatment platform for patients who are difficult to treat due to antibiotic resistance.

## Electronic supplementary material

Below is the link to the electronic supplementary material.

Supplementary Material 1

## Abbreviations

MRSA: Methicillin-resistant Staphylococcus aureus
MDR: Multi Drug Resistant
ZnO: Zinc Oxide
UV: Ultraviolet
Au/ZnO: Gold-Zinc Oxide bimetal
Con A: Concanavalin A
Au/ZnO-Con A: Con A conjugated Au/ZnO
LPS: Lipopolysaccharide
CTAB: Cetyl trimethyl ammonium bormide
DW: Distilled water
TEM: Transmission electron microscope
EDS: Energy dispersive X-ray spectroscopy
XRD: X-ray diffractometer
DLS: Dynamic Light Scattering
NTA: Nanoparticle Tracking Analysis
PFA: Paraformaldehyde
DPBS: Dulbecco’s Phosphate-Buffered Saline
FBS: Fetal bovine serum
CCK-8: Cell Counting Kit-8
FTIR: Fourier transform infrared
BSA: Bovine serum albumin
DMSO: Dimethyl sulfoxide
PI: Propidium iodide
NPs: Nanoparticles
