# Supplementary material for: Photocatalytic effect of gold-zinc oxide composite nanostructures for the selective and controlled killing of antibiotic-resistant bacteria and the removal of resistant bacterial biofilms from the body
Source: Nano Converg. 2025 May 14;12:23. doi: 10.1186/s40580-025-00488-z (PMC12078748; doi:10.1186/s40580-025-00488-z)
Supplement: Supplementary file 1 — Supplementary Material 1 [file 40580_2025_488_MOESM1_ESM.docx]

**Supplementary data**

Supplementary data can be accessed online.

**
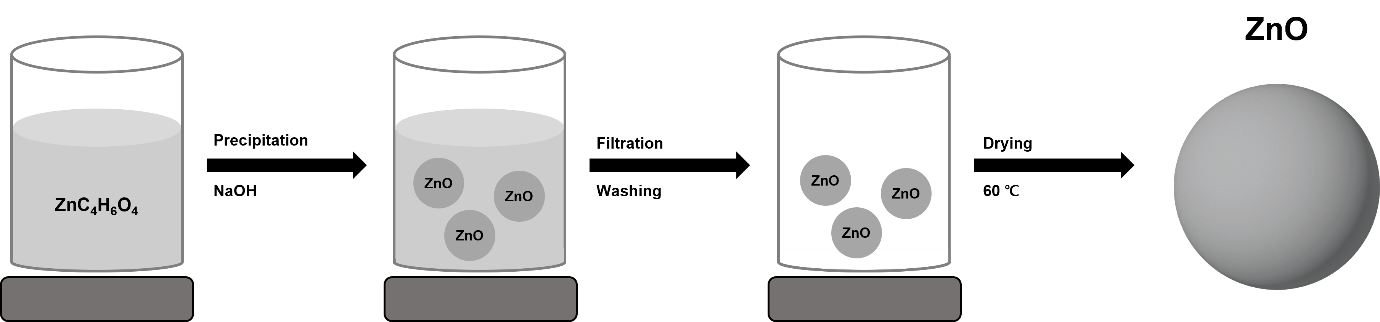
**

**Figure S1.** Schematic illustration of ZnO nanoparticle synthesis via precipitation method.

**
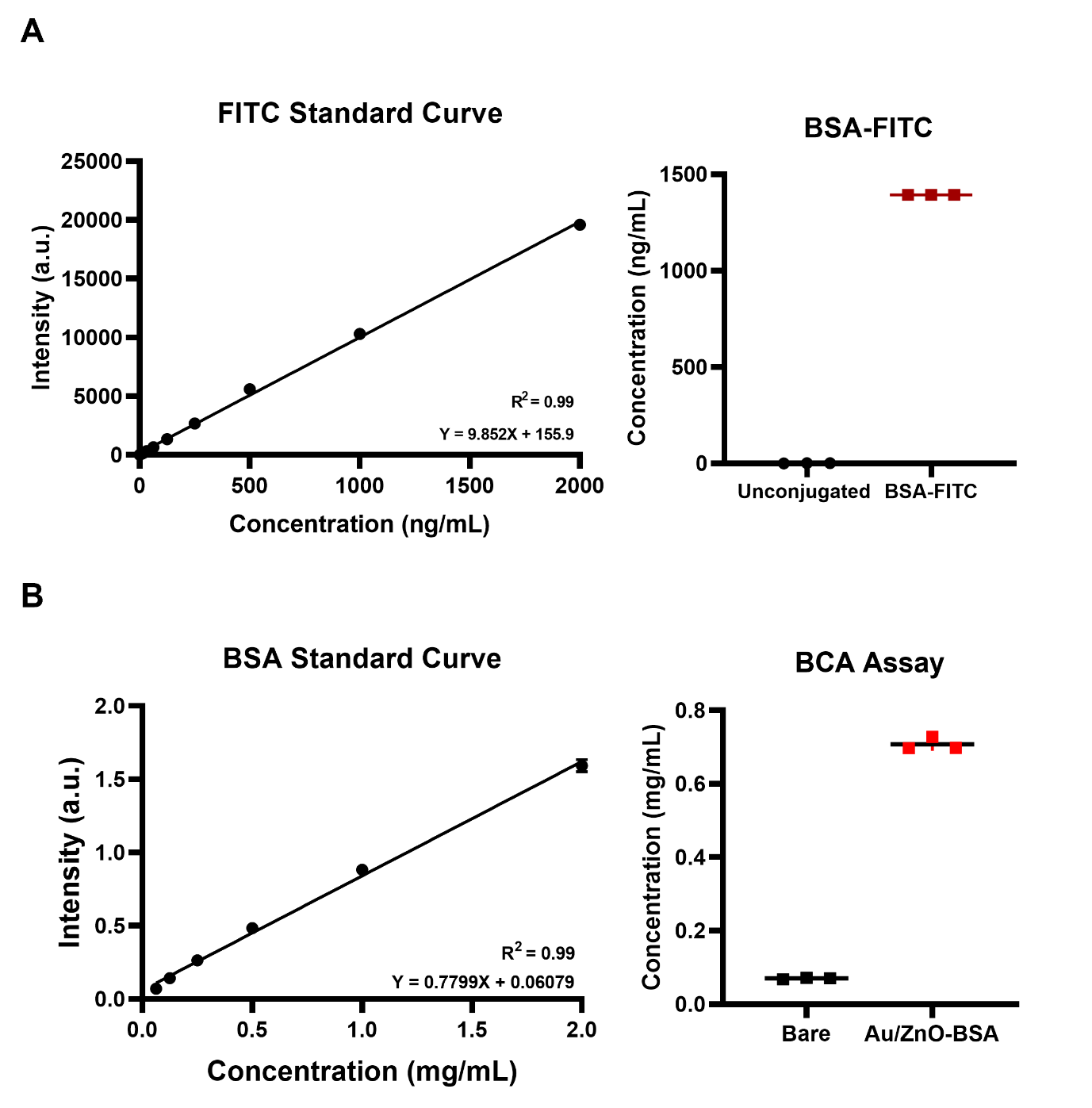
**

**Figure S2.** Au/ZnO-BSA Characterization. (A) FITC Standard curve and conjugation results for the BSA-FITC fluorescent dye analysis. (B) BSA protein standard curve for the BCA assay, and BSA protein concentration calculated.

**
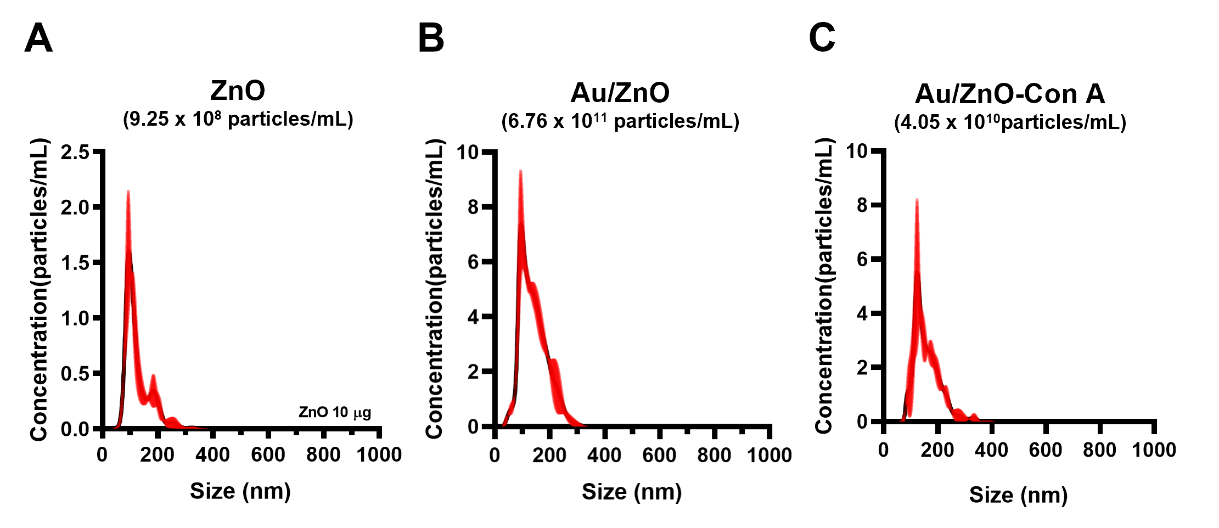
**

**Figure S3.** Particle concentrations calculated by nanoparticles tracking analysis (NTA). Concentrations of (A) 10 µg of ZnO nanoparticles and synthesized (B) prepared Au/ZnO nanoparticles and (C) prepared Au/ZnO-Con A nanoparticles.


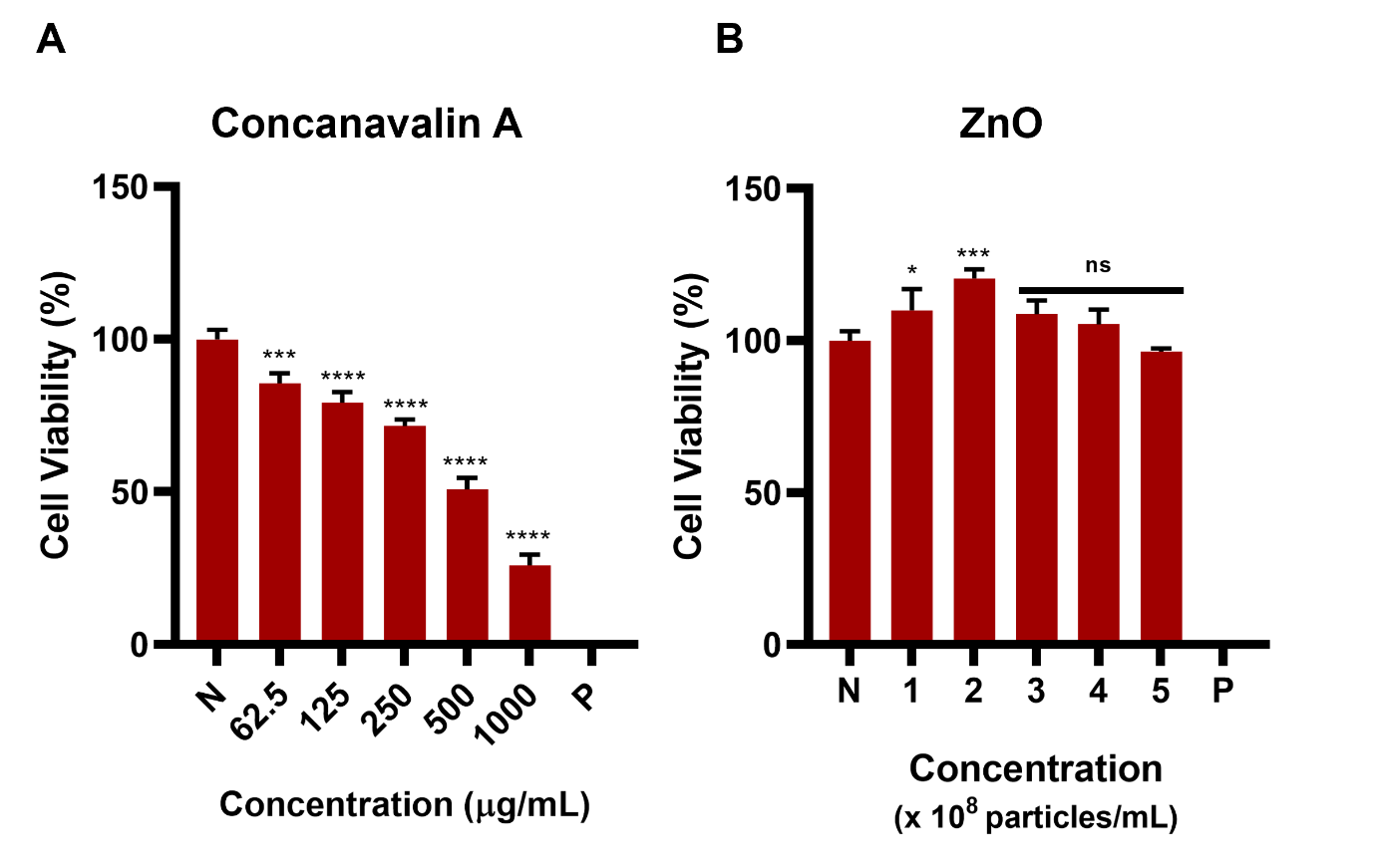


**Figure S4.** Cytotoxicity evaluation using a Cell Counting Kit-8 (CCK-8). Cell viability evaluations for (A) Concanavalin A and (B) ZnO nanoparticles.

**Supplementary Materials**


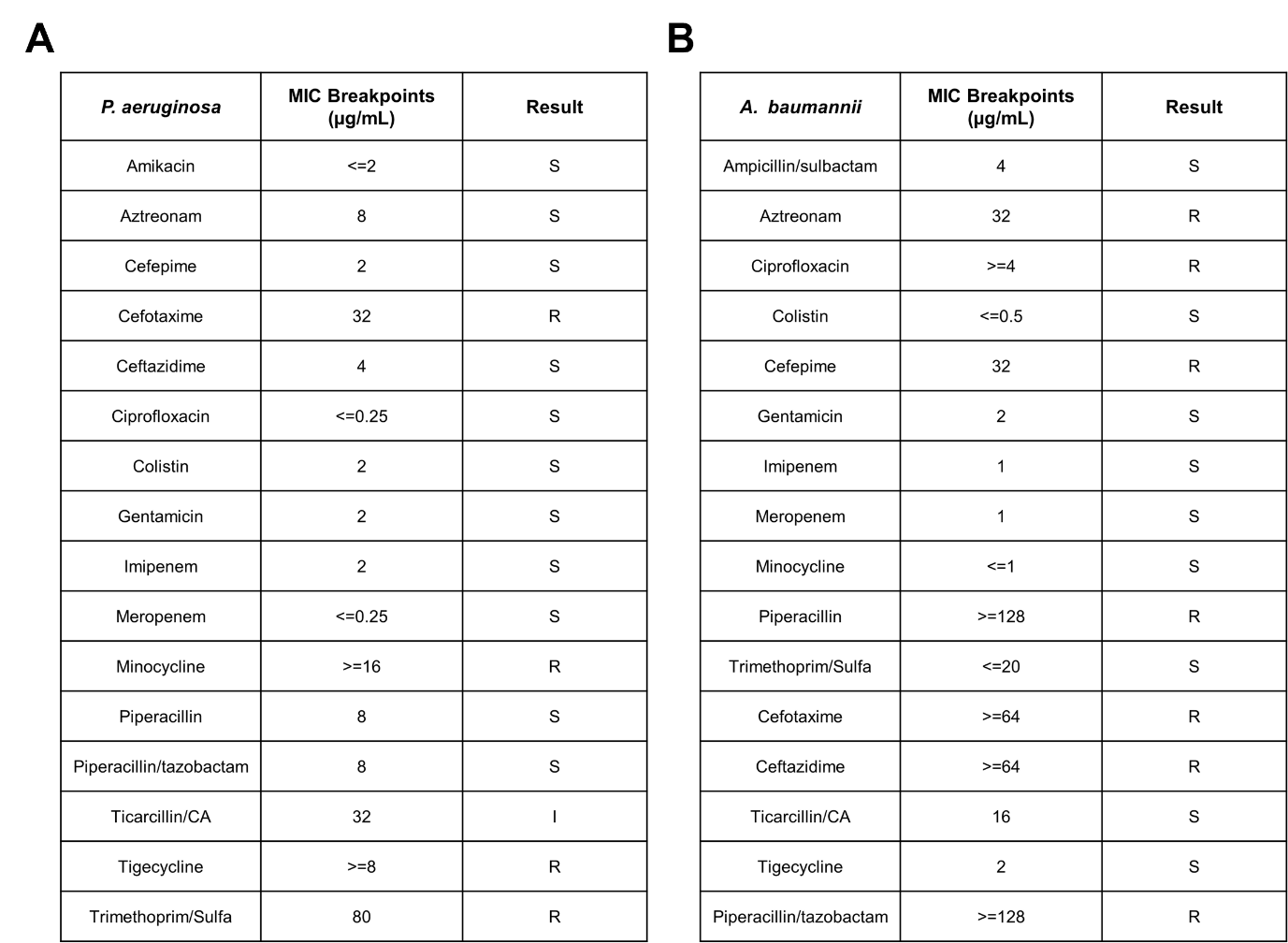


**Table S1.** Antibiotic susceptibility tests. Antibiotic susceptibility tests were performed on (A) *P. aeruginosa* and (B) *A. baumannii* bacterial strains using Vitek2 (Biomérieux, SA). “S” indicates susceptible, “I” indicates intermediate, and “R” indicates resistant. The results were provided by Chung-Ang University Hospital.
